# Small-Animal PET Imaging of Amyloid-Beta Plaques with [^11^C]PiB and Its Multi-Modal Validation in an APP/PS1 Mouse Model of Alzheimer's Disease

**DOI:** 10.1371/journal.pone.0031310

**Published:** 2012-03-09

**Authors:** André Manook, Behrooz H. Yousefi, Antje Willuweit, Stefan Platzer, Sybille Reder, Andreas Voss, Marc Huisman, Markus Settles, Frauke Neff, Joachim Velden, Michael Schoor, Heinz von der Kammer, Hans-Jürgen Wester, Markus Schwaiger, Gjermund Henriksen, Alexander Drzezga

**Affiliations:** 1 Nuklearmedizinische Klinik und Poliklinik, Klinikum rechts der Isar, Technische Universität München, Munich, Germany; 2 Evotec AG, Hamburg, Germany; 3 Neurologische Klinik und Poliklinik, Klinikum rechts der Isar, Technische Universität München, Munich, Germany; 4 Institut für Pathologie - Biomedizinische Mikroskopie, Helmholtz Zentrum München, Neuherberg, Germany; 5 Institut für Röntgendiagnostik, Klinikum rechts der Isar, Technische Universität München, Munich, Germany; 6 Institut für Allgemeine Pathologie und Pathologische Anatomie - Neuropathologie, Klinkum rechts der Isar, Technische Universität München, Munich, Germany; 7 TaconicArtemis GmbH, Cologne, Germany; National Institutes of Health, United States of America

## Abstract

In vivo imaging and quantification of amyloid-β plaque (Aβ) burden in small-animal models of Alzheimer's disease (AD) is a valuable tool for translational research such as developing specific imaging markers and monitoring new therapy approaches. Methodological constraints such as image resolution of positron emission tomography (PET) and lack of suitable AD models have limited the feasibility of PET in mice. In this study, we evaluated a feasible protocol for PET imaging of Aβ in mouse brain with [^11^C]PiB and specific activities commonly used in human studies. In vivo mouse brain MRI for anatomical reference was acquired with a clinical 1.5 T system. A recently characterized APP/PS1 mouse was employed to measure Aβ at different disease stages in homozygous and hemizygous animals. We performed multi-modal cross-validations for the PET results with ex vivo and in vitro methodologies, including regional brain biodistribution, multi-label digital autoradiography, protein quantification with ELISA, fluorescence microscopy, semi-automated histological quantification and radioligand binding assays. Specific [^11^C]PiB uptake in individual brain regions with Aβ deposition was demonstrated and validated in all animals of the study cohort including homozygous AD animals as young as nine months. Corresponding to the extent of Aβ pathology, old homozygous AD animals (21 months) showed the highest uptake followed by old hemizygous (23 months) and young homozygous mice (9 months). In all AD age groups the cerebellum was shown to be suitable as an intracerebral reference region. PET results were cross-validated and consistent with all applied ex vivo and in vitro methodologies. The results confirm that the experimental setup for non-invasive [^11^C]PiB imaging of Aβ in the APP/PS1 mice provides a feasible, reproducible and robust protocol for small-animal Aβ imaging. It allows longitudinal imaging studies with follow-up periods of approximately one and a half years and provides a foundation for translational Alzheimer neuroimaging in transgenic mice.

## Introduction

Neuritic plaques containing Aβ and neurofibrillary tangles continue to define the neuropathological entity of AD and a definite diagnosis can still only be established post-mortem [1]–[4]. The increased production of certain Aβ species, their aggregation and deposition as insoluble plaques is regarded as an early and key pathology in the development of AD, and many modern treatment approaches are directed at the prevention or reversal of Aβ plaque deposition in the brain [5]. Aβ plaque imaging with PET has now entered the realm of the revised criteria for diagnosis of Alzheimer's disease [6] and helps to further improve early and specific diagnosis and treatment monitoring [7].

Several radiolabeled compounds with high affinity and specificity for Aβ aggregates have been developed [8]–[19]. Among these compounds, [^11^C]6-OH-BTA-1 ([^11^C]PiB) is presently the one most extensively evaluated worldwide.

Advances in PET technology have facilitated the imaging of small animals [20]–[22]. Transgenic mice possessing the mutations held responsible for familiar AD have been shown to develop Aβ deposits, tangles and synaptic dysfunction, thus, mimicking human AD pathology [23]–[31]. However, the correspondence between preclinical and clinical data on Aβ imaging remains a challenge in transgenic models of AD [32].

Earlier in vivo, in vitro and ex vivo analyses suggested that [^11^C]PiB shows specific binding to Aβ plaques in transgenic mice [33]. Also, high-resolution imaging studies, such as MRI [34], [35] and in vivo optical imaging [36], [37] demonstrated specific binding to Aβ plaques in transgenic mouse models. Although small-animal PET imaging could allow for the quantification of global Aβ plaque load in the brain in vivo, previous studies suggest that detection of small differences between transgenic and healthy control animals by PET remains a challenge [33], [38], [39]. This may be due to methodological limitations like image resolution in relation to the small size of target structures, image co-registration, animal motion, signal-to-noise ratios and cranial tracer distribution in rodents. Furthermore, transgenic mouse models suitable for PET imaging of Aβ plaques were lacking [40]. Only one study showed in vivo mouse brain imaging using PET with [^11^C]PiB [41], though very high specific activities of the tracer were required to obtain a signal.

Here, we report the evaluation and multi-modal cross-validation of a feasible small-animal PET imaging approach with [^11^C]PiB. Specific binding of [^11^C]PiB to Aβ plaques in transgenic AD mouse brain could be demonstrated in PET using specific activities as used in clinical routine for humans. The mouse study collective was designed with three transgenic groups of an APP/PS1 mouse model [31] which serve as examples for different AD stages. PET distinguished animals according to their Aβ plaque burden and these in vivo findings were validated in all other experimental modalities.

## Results

### Mouse brain PET with [^11^C]PiB

For in vivo assessment of cerebral Aβ plaque deposition, 47 animals in five study groups were scanned at least once with [^11^C]PiB. 35 of these animals also received an in vivo MR brain scan on a human clinical scanner (Table 1).

**Table 1:** Mouse study collective and numbers of mice per experiment.

study group	sub-group	age [months]	weight [g]	PET	injected dose [MBq]	MRI	CT	autoradio	histological quantification		ELISA	biodistribution		binding assay
									Thio-flavin S	Aβ 40/42		brain	cranium	
**tg-old**	5 ♀	23.2±0.1	24.5±1.2	5	13.9±3.1	5		5	5	5	2			
	2 ♀	29.1±0.0									2			1
**tgtg-young**	5 ♀	9.2±0.0	22.4±0.5	5	28.9±7.9	5		5	5	5	2			
	4 ♂	9.4±0.1	27.1±0.5	4	32.3±4.6	1								
	5 ♂	9.0±0.0										5	5	
	2 ♀	9.1±0.1	25.3±2.1	2	63.4±3.7		2	2	2	2	2	2		
	3 ♀	9.3±0.0									3			1
**tgtg-old**	4 ♀	21.1±0.1	23.3±0.6	5	22.4±2.6	4		4	5	5	4	1	1	
	4 ♀	21.6±0.0	21.9±0.9	4	32.0±2.7						4	4	4	
	3 ♀	18.7±0.0									3			1
**ctl-young**	5 ♀	9.4±0.1	25.6±0.8	5	15.8±1.9	5		2	5	1				
	5 ♂	9.0±0.0	31.0±0.3	5	25.5±3.3	5								
	5 ♀	9.3±0.0										5	5	
	2 ♀	9.0±0.6	32.7±1.0	2	51.2±1.4		2	2	2	2		2		
**ctl-old**	5 ♀	23.6±0.1	29.8±1.9	5	20.7±3.3	5		4	5	2				
	5 ♂	23.0±0.0	33.3±1.5	5	11.9±3.1	5								
	5 ♂	23.2±0.0										5	5	
	1 ♀	29.8												1
**SUM**				47		35	4	24	29	22	22	24	20	4

Five study groups were defined, three of them with transgenic APP/PS1 mice, the others with age- and gender-matched controls. Major subgroups were female. *Old* refers to an age of about 23 (hemizygous (*tg*)) and 21 months (homozygous (*tgtg*)). *Young* is defined as an age of 9 months. Young homozygous study group (*tgtg-young*) and both control groups (*ctl*) were designed to reveal possible gender effects. As an overview and orientation for this study, numbers in each cell state how many animals per subgroup were analyzed in the corresponding experiment. Mean ages, weights and injected doses are shown for each subgroup including standard deviation. The pairwise correlations of these modalities are shown in Figure 7 and Figure S10.

(tg: hemizygous APP/PS1, tgtg: homozygous APP/PS1, ctl: C57BL6/J control animals).

**Figure 1:**
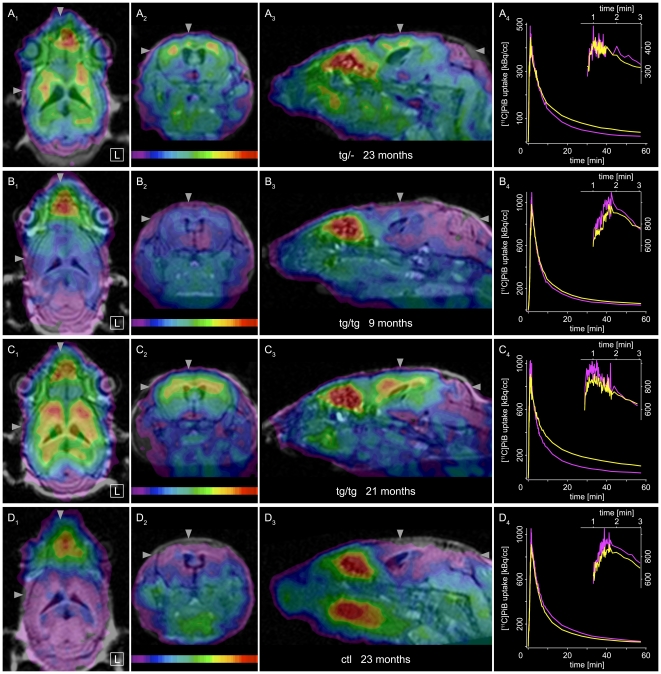
Small-animal [^11^C]PiB PET/MRI overview. [^11^C]PiB PET co-registered to in vivo 1.5T cranial MRI of the same mouse. Overview of cranial tracer uptake shows images of four representative animals from the major study groups in radiological orthogonal perspective (20–30 min frame). (**A**) 23 month old female hemizygous APP/PS1 mouse (weight: 20.8 g, injected dose: 14.7 MBq, color scale 37–144 kBq/cc), (**B**) 9 month old female homozygous APP/PS1 mouse (weight: 22.2 g, injected dose: 15.2 MBq, color scale 60–350 kBq/cc) (**C**) 21 month old female homozygous APP/PS1 mouse (weight: 24.5 g, injected dose: 24.2 MBq, color scale 73–280 kBq/cc), (**D**) 23 month old female C57BL/6J control mouse (weight: 29.9 g, injected dose: 15.1 MBq, color scale: 66–300 kBq/cc). *Columns* from left to right show horizontal (**1**), coronal (**2**) and sagittal (**3**) views. The *right column* (**4**) shows corresponding neocortical (*yellow*) and cerebellar (*magenta*) time-activity curves (TACs). *Inset* (**5**) shows initial tracer dynamics on a smaller time scale (1 to 3 min) to delineate the peak of uptake required for quantification of PET data. Difference between transgenic and control animals is significant for each study group visibly and analytically. For the young homozygous animal, it is seen in the lower color scale range. Cortex in B_2_ shows uptake towards *blue* and *cyan*. Same structures show lowest uptake in D_2_ (*magenta*, corresponding to cerebellum). TACs confirm visual perception: neocortex TAC in B_4_ intersects cerebellum TAC and stays above it (neocortex-to-cerebellum ratio >1) while neocortex TAC in D_4_ remains below the cerebellum TAC (ratio <1). PET color look-up-table is *UCLA* (Pmod) with lower thresholds set to still visualize the cerebellum. Arrowheads indicate slice positions. Slice coordinates (corresponding to Paxinos atlas) are: horizontal Bregma −1.90 mm, coronal Bregma −0.10 mm and sagittal 0.65 mm lateral. Image scale is double size of reality. Further results for these animals are shown in Figure 7.

Visual inspection of co-registered PET/MR images revealed distinct activity retention in the cortex of all transgenic mice corresponding to their study group, whereas for all control animals the cortex appeared to be free of specific activity uptake (Figure 1). In transgenic mouse brain the activity uptake expanded throughout the entire cortex, with slightly stronger signal in frontal neocortical compared to hippocampal regions and a stronger signal in the thalamus. These findings are in good correspondence to the earlier onset of plaque deposition in cortex and to large plaque sizes in thalamus as observed by Willuweit et al. [31] ex vivo.

Time-activity curves (TACs) of target and reference tissues showed characteristics that were common for all study groups (Figure 1 and Figure S6). Initial cerebellar uptake was always higher than initial cortical uptake and cortical TACs of control animals fell below cerebellar TACs early. In contrast, for each transgenic animal the cortical TAC showed higher values than the cerebellar TAC from around 3 min p.i. and remained distinctly separable. From about 10 min p.i. on, each transgenic animal could be assigned to its study group by its neocortex-cerebellum TACs.

**Figure 2:**
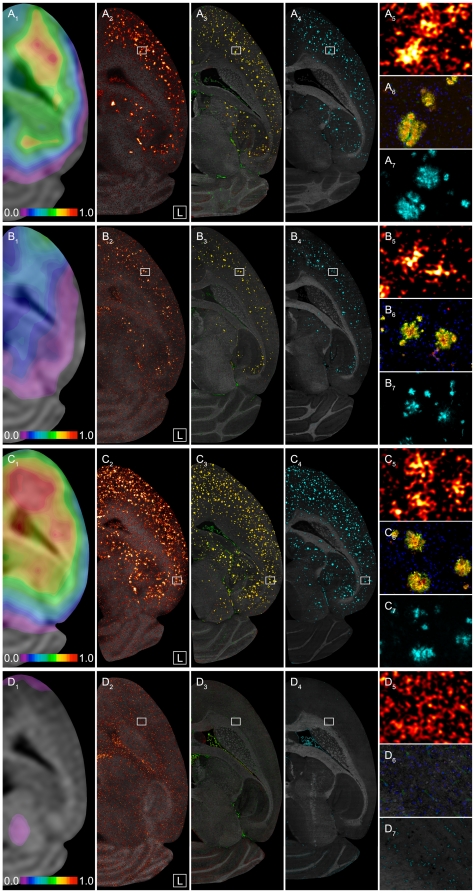
Mouse Aβ plaque pathology in vivo and ex vivo. PET binding potential maps for [^11^C]PiB and corresponding autoradiography and fluorescence microscopy images of neighboring horizontal brain sections showing data from the same animals presented in Figure 1. Left brain halves are shown. Frontal cortex is at top and cerebellum at bottom of each panel. (**A**) 23 month old female hemizygous APP/PS1 mouse, (**B**) 9 month old female homozygous APP/PS1 mouse, (**C**) 21 month old female homozygous APP/PS1 mouse, (**D**) 23 month old female C57BL/6J control mouse. *Column* (**1**): Binding potential maps for [^11^C]PiB (BP_ND_, MRTM2) matched to MRI. Shown is the same horizontal level as in Figure 1 and S1. Color table is *UCLA* (Pmod). Width of color scale represents 3 mm in reality. *Column* (**2**): Digital [^3^H]PiB ex vivo autoradiograph with optical image (*gray*) of a brain section of the same animal, killed 1 hour p.i.. Color table is *Red Hot* (ImageJ). *Column* (**3**): Double immunofluorescence microscopy for Aβ40 (*green*) and Aβ42 (*red*). Anatomical reference (*gray*) is provided by control channel (Cy3). Column (**4**): Thioflavin S fluorescence (FITC excitation, *cyan*). Anatomical reference (*gray*) is provided by DAPI fluorescence. *Right column*: Identical Aβ plaque constellations of adjacent sections (as marked by white rectangle in columns (**1**) to (**3**). *Top panel* (**5**): magnification of digital autoradiograph as seen in *column* (**2**). *Middle panel* (**6**): corresponding magnified view of Aβ40/Aβ42 stain as seen in *column* (**3**). *Bottom panel* (**7**): corresponding magnified view of Thioflavin S stain as seen in column (**4**). Columns (**2**) to (**4**) show directly neighboring 10 µm thick sections of the left brain half from bottom to top of skull at about 1.9 mm below Bregma. Width of zoom panels in rightmost column represents 350 µm in reality. Complete orthogonal views for binding potential maps are shown in Figure S1.

The individual in vivo radioligand binding was examined by calculating the binding potential with a reference tissue approach using the cerebellum [41]–[44]. Parametric images of regional Aβ plaque burden for representative animals were created with the 2-step multilinear reference tissue model 2 (MRTM2, [43], [45]) as shown in Figure 2 and Figure S1. The binding potential values for neocortex estimated by the same model for the whole study collective showed highly significant separation of all transgenic animals from controls and a clear distinction of AD animals belonging to different study groups (Figure S10). Old homozygous AD animals (tgtg-old) exhibited highest activity retention, followed by old hemizygous (tg-old) and young homozygous mice (tgtg-young). The tg-old animals were at all scans in between the homozygous groups while their results were generally closer to those of the tgtg-young group. In control animals we never observed any specific tracer uptake within the entire brain.

In general, no significant differences in activity uptake were found between old and young or female and male control animals. Further, no significant difference was found between male and female young homozygous animals. Also, there were no differences between right and left hemispheric tracer uptake observed in all animals.

The robustness and consistency of PET results was shown by performing test-retest experiments (Figure S5), by calculating alternative measures for radioligand binding (Table S1) and by averaging all neocortical and all cerebellar TACs for each study group (Figure S6).

In general, the results above show a tight correlation of visual inspection and PET analysis with all other modalities (Figure 2 and Figure S10).

### Autoradiography with [^3^H]PiB ex vivo

Extensive ex vivo autoradiography of brain slices was performed to verify that the cortical tracer uptake values as measured by PET represent true binding of [^11^C]PiB to cortical Aβ plaques. All animals in this analysis had a PET scan with [^11^C]PiB, before.

On visual inspection, representative slices showed a homogenously dotted pattern of intensive multi-focal tracer retention throughout the cortex of transgenic mice with a fully symmetric right-left appearance. Particularly high uptake was detected in the neocortex, hippocampus and thalamus (Figure 2 (column 2)). The strong uptake in the thalamus was notable due to very high tracer retention in fewer but much larger plaques. Plaques were also present in the olfactory bulb although smaller in size (data not shown). Without exception, the cerebellum was free of specific [^3^H]PiB uptake in autoradiography. The entire brain of control animals did neither show focal nor diffusely increased neocortical tracer uptake (Figure 2). A clear difference in the amount of [^3^H]PiB uptake, corresponding to different stages of Aβ plaque burden, was noted even visually. Hence, the representative samples of the four major study groups showed corresponding results of ex vivo [^3^H]PiB uptake in autoradiography to [^11^C]PiB uptake as measured in PET. In addition, individual uptake patterns were in full correspondence to the patterns of Thioflavin S and anti-Aβ40/42 stains done on neighboring sections (Figure 2).

Visual perception of differences in ex vivo tracer uptake between the study groups (Figure 2) could be confirmed quantitatively by measuring a total of 64 slides from 24 animals of the study collective yielding 248 observations for the cortical region (Table 1).

The neocortex-to-cerebellum ratios of [^3^H]PiB uptake fully reflected the in vivo PET findings for the same target region. The average ratios for tg-old were 1.90±0.26 (range: 1.46–2.11), for tgtg-young 1.25±0.07 (range: 1.19–1.33), for tgtg-old 2.54±0.27 (range: 2.13–2.71) and for ctl-old 0.93±0.02 (range: 0.90–0.95). Statistical significance of differences between groups was tested for ctl-old against tgtg-young (p<0.001), tgtg-young against tg-old (p = 0.004) and tg-old against tgtg-old (p = 0.002) corresponding to the staging of Aβ load in these groups.

No significant differences in tracer uptake were found between right and left neocortical regions in all animals and between old and young control animals.

The individual results and pairwise correlations (Figure S10) are reported below.

### Regional brain biodistribution of [^11^C]PiB

Twenty animals from the homozygous transgenic study groups and matched controls were used for ex vivo regional brain biodistribution of [^11^C]PiB at 30 min p.i.. Mouse brains were dissected into four regions: 1. telencephalon as the major target region, 2. olfactory system for its proximity to high extracerebral uptake regions, 3. cerebellum as the reference region and 4. diencephalon and midbrain as the remaining brain structures. The cerebellum was used as the reference region for ratio calculations of individual %ID/g values.

The target-to-reference ratios for telencephalon confirmed the in vivo PET measurements for neocortex in these study groups. The results for the old homozygous animals showed a large difference to the young homozygous mice (p<0.001). This corresponded to the large differences between these two groups as seen in all the other experimental modalities (Table 2).

**Table 2:** Regional brain biodistribution of [^11^C]PiB.

study group	olfactory system	telencephalon	diencephalon and midbrain
**tgtg-young**	1.27±0.24	1.87±0.58	1.79±0.57
**tgtg-old**	1.90±0.22	4.23±0.46	2.22±0.47
**ctl-young**	0.87±0.23	0.82±0.09	1.10±0.17
**ctl-old**		0.67±0.12	1.11±0.13

**Figure 3:**
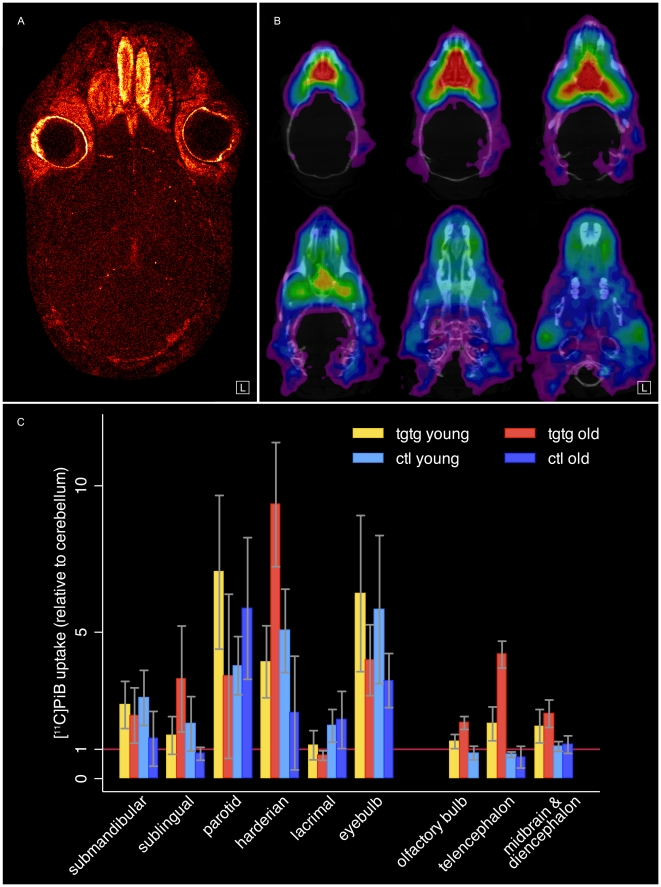
Extracerebral tracer retention. High [^11^C]PiB uptake in regions frontal to the brain were accurately validated to be extracerebral. (**A**) Cranial [^3^H]PiB ex vivo autoradiography. 15 µm thick section of a complete mouse head showing exact anatomical locations of unspecific tracer retention (male tgtg, 16 month old). Exposition time needed to be shortened to achieve good resolution of extracerebral tissues. For this reason, only few plaques can be seen in the brain. Color table: *Red Hot* (ImageJ) (**B**) CNS removal during [^11^C]PiB PET. 9 month old male homozygous APP/PS1 mouse was scanned in vivo for 30 min before the complete brain was extracted and scanned for further 30 min together with the skull. The skull of the ex vivo [^11^C]PiB PET scan is co-registered to a cranial CT for better orientation and shown on six horizontal slices which are 1 mm apart (top left horizontal level at about −1.9 mm Bregma in correspondence to all other figures). Both parotid glands can be seen on bottom section. Color table is *UCLA* (Pmod) (**C**) Ex vivo biodistribution of [^11^C]PiB relative to cerebellar uptake in (extracerebral) glandular tissues and eyebulbs in both homozygous and both control study groups. Cerebral biodistribution data from the same animals as presented in Table 2 is included graphically as reference. Data show that olfactory bulb does not contribute to high surrounding uptake in harderian glands and eyebulbs. Column heights represent means, error bars represent standard deviation.

Mouse brain was dissected into four regions (olfactory system, telencephalon, cerebellum and remaining brain structures) 30 min p.i.. Results show mean [^11^C]PiB uptake ratios (± SD) of the three target regions relative to cerebellum (initially measured as %ID/g) for the homozygous study groups and both control groups. Data are reported graphically in Figure 3 as reference to extracerebral [^11^C]PiB distribution.

The young transgenic animals could easily be separated from the controls (p = 0.012). Differences between the young and old control groups were not significant (p = 0.090). In the control groups, it is notable that the tracer uptake for the telencephalical region relative to cerebellum was reversed (<1).

A similar behavior of relative uptake of [^11^C]PiB was found in the other two target regions. The PiB uptake in the olfactory system in transgenic mice is specific, but considerably lower than in cortical regions. Young transgenic animals even had no significantly higher uptake than young controls (p = 0.491) corresponding to a low Aβ plaque load in the olfactory bulb at younger ages. The relative tracer uptake in old homozygous animals was slightly higher than in young ones (p = 0.027) but by far not as distinct as for the telencephalon. This confirmed previous reports [31] and is consistent with our observations of smaller and fewer plaques in the olfactory bulb. This result was also notable in the context of unspecific tracer binding. The olfactory bulb reaches in between extracerebral structures with high unspecific tracer uptake but was not a contributing region for these high uptakes.

Relative [^11^C]PiB uptake of the remaining basal brain structures (diencephalon and midbrain) was already significantly higher in young transgenic animals than in controls (p = 0.031)) (Table 2). However, in old homozygous animals it was not significantly higher to the young ones (p = 0.318). In our experience and in consistency with previous reports in the same or similar models [31], [46], [47], the neuroanatomical structure with the major contribution to uptake in this region was the thalamus. The difference between young and old control groups was not significant (p = 0.114). For this region, it is notable that the relative tracer uptake was not reversed in the control groups (>1). The relative [^11^C]PiB uptake behavior is also shown graphically in Figure 3.

### Extracerebral tracer retention in proximity to brain

We observed considerable [^11^C]PiB retention in regions of the mouse head that appeared to be extracerebral, possibly around nasal and eye cavities, but very close to the brain (Figure 1). To distinguish specific PiB uptake in brain from probably unspecific extracerebral uptake and for further validation of our PET imaging and co-registration protocol, we performed variants of the general in vivo and ex vivo experiments.

Sequential [^11^C]PiB/[^18^F]FDG PET for five old homozygous and five control animals while keeping the animal in place provided automatic overlay of both PET images and clear spatial localization of the brain. Thus, it was possible to confirm that the high frontal [^11^C]PiB retention was indeed located outside the brain (Figure S2).

Additionally, the in vivo PET protocol was modified to a two-step in vivo/ex vivo PET protocol, in which complete heads without brains of four male tgtg-young and four ctl-young animals were scanned from about 35 min to 65 min p.i. (Figure 3B). The remaining [^11^C]PiB retention in exclusively extracerebral anatomical structures clearly shows the same uptake pattern as seen in PET in vivo.

To confirm the findings from in vivo and ex vivo PET, the 20 animals from the regional brain biodistribution study (Table 2) were also used to measure [^11^C]PiB uptake in various cranial organs (Figure 3C). The organs with the most prominent [^11^C]PiB uptake were the harderian and parotid gland and the eyebulbs. In general, tracer retention varied unsystematically between animals of the same groups and no differences could be detected between transgenic and control animals.

To further validate unspecific extracerebral tracer retention, ex vivo [^3^H]PiB autoradiographs of a complete transgenic homozygous mouse head showed the exact locations of unspecific tracer retention in various anatomical structures very accurately (Figure 3A). Exposition time needed to be shortened to achieve good resolution of extracerebral tissues. For this reason, only few plaques can be seen. The analogy of the unspecific extracerebral uptake pattern in ex vivo [^3^H]PiB autoradiography and ex vivo [^11^C]PiB PET of the head can be seen well.

As the olfactory bulb reaches in between the anatomical structures that have been characterized with high unspecific tracer uptake, it was included in [^11^C]PiB regional brain biodistribution, [^3^H]PiB autoradiography, Thioflavin S and Aβ40/42 histological analyses whenever possible. In general, it showed smaller and fewer plaques and lower uptake values, confirming that it was not involved in the higher tracer retention regions around it.

The spectrum of results, above, validated the high unspecific tracer uptake to be extracerebral. The proximity of frontal brain parts to extracerebral anatomical structures, in particular present within the eye cavities, confirmed the importance of very accurate image co-registration for reliable PET analyses described above. The principle and high quality of our image co-registration method is presented in Figure S3.

### Thioflavin S and Aβ40/42 antibodies for plaque quantification

Brain sections were stained with Thioflavin S (29 animals) and with double immunofluorescence against Aβ_x–40_ (anti-Aβ40) and Aβ_x–42_ (anti-Aβ42) (22 animals) for the histological quantification of Aβ plaque load and plaque size distribution (Table 1) by applying a computerized image analysis and object recognition algorithm similar as reported, previously [31].

**Figure 4:**
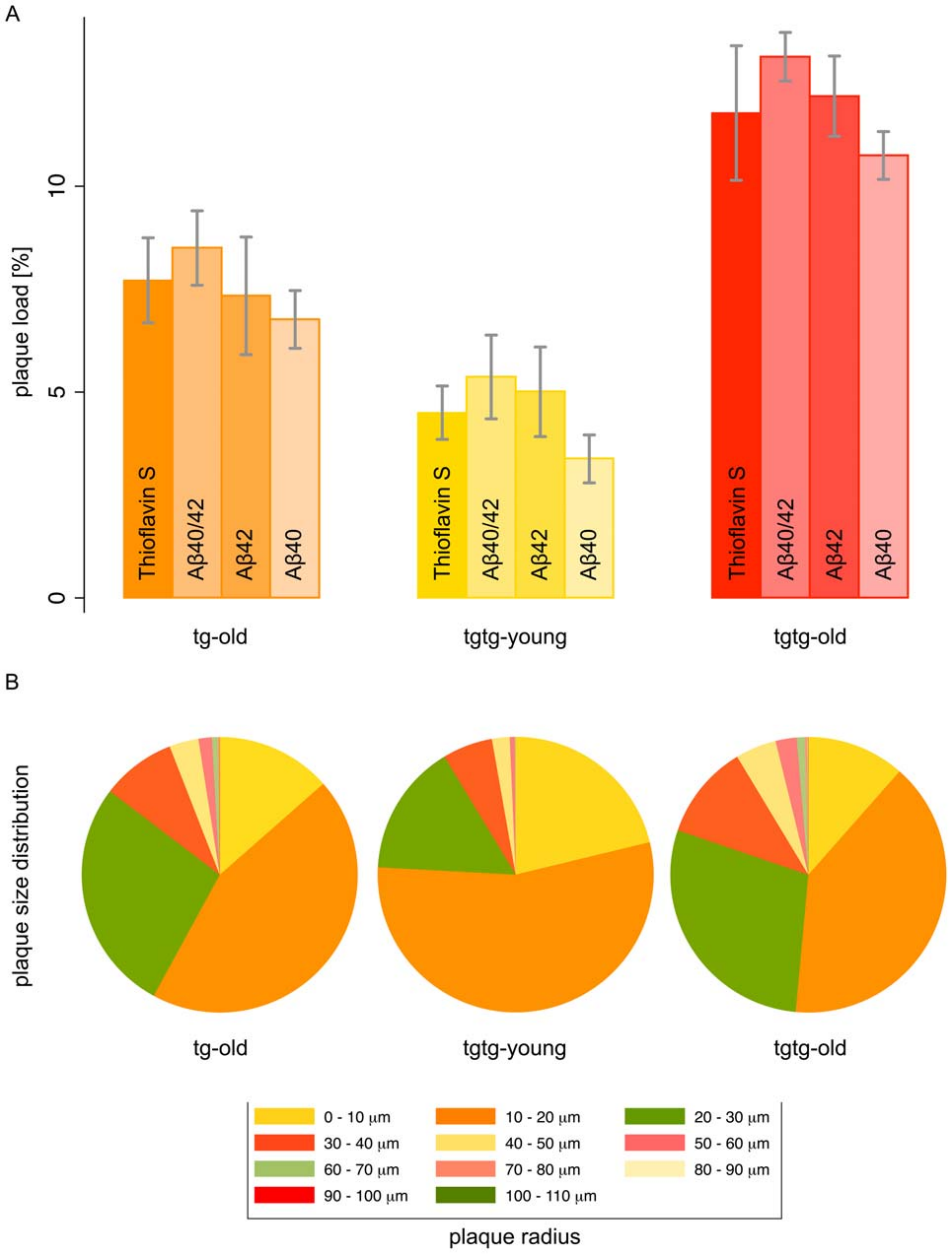
Histological Aβ plaque burden and plaque size distribution in neocortex. Aβ plaque burden and size of individual plaques were analyzed on histological sections stained with Thioflavin S and double immunofluorescence against Aβ40 and Aβ42 by applying a semi-automatic imaging algorithm. All animals were analyzed in PET, before. Shown here, are the results for neocortex of the transgenic study groups: tg-old (*orange*), tgtg-young (*yellow*) and tgtg-old (*red*). (**A**) Aβ plaque burden of each transgenic group as measured by Thioflavin S, compound anti-Aβ40/42, anti-Aβ42 and anti-Aβ40. Compound anti-Aβ40/42 result shows co-localization of both Aβ species. (**B**) Plaque size distribution in each transgenic study group. Here, the anti-Aβ42 signal was used for its highest signal-to-noise. Its strong association with the Thioflavin S signal is shown in Figure S7.

Thioflavin S has not been used with the applied semi-automated method of Aβ plaque quantification in this mouse model, before [31]. To validate Thioflavin S staining as a robust and comparably easy method for Aβ plaque quantification we first analyzed the relation of Thioflavin S sensitivity to anti-Aβ40/42 sensitivities for Aβ plaque detection in a pairwise manner. The correlations between Thioflavin S quantification with each of the antibodies and with their compound signal are presented in Figure S7. In addition, Thioflavin S recognized a similar amount of relative Aβ plaque load as the anti-Aβ40/42 compound signal (Figure 4A) which seems to parallel the comparable affinities of PiB for both Aβ species [18]. Furthermore, the anti-Aβ40/42 compound result (Figure 4A) reflects that the anti-Aβ40 and anti-Aβ42 measurements are mostly co-localized.

Thioflavin S staining was excellent for analyzing tissues with Aβ deposits. However, it showed some unspecific binding in Aβ-free regions depending on the neuroanatomical location, in contrast to the specific Aβ antibodies. Among the regions we have measured, the highest unspecific values were found in thalamus of control animals. We expect this to be mostly related to the texture of the regional brain tissue. It may also be method-related as exposition and measurement parameters were kept identical for each stain and were adjusted to measurements in the neocortex.

### Aβ plaque area and plaque size distribution

Binding of [^11^C]PiB to Aβ loaded brain regions is probably influenced both by total plaque volume as well as by individual plaque size and type [35], [48]. Hence, we measured relative plaque areas and plaque size distributions using a large dataset for a robust analysis of neocortex with Thioflavin S (643 observations) and Aβ40/42 antibodies (158 observations) (Figure 4 and Figure S7).

The results from histological Aβ plaque quantification in neocortex also were consistent with in vivo PET results for the same region. Highest values were observed for the tgtg-old group, followed by tg-old and tgtg-young (see Figure S10 for pairwise correlations).

The results of plaque load as measured by Thioflavin S are reported, here. Their values in target regions with Aβ deposits were representative for the analysis with Aβ40/42 antibodies as shown above (Figure S7). Plaque load based on Thioflavin S binding in neocortex was measured for tg-old as 7.72±1.03% (range: 6.27–9.07%), for tgtg-young as 4.68±0.70% (range: 3.53–5.37%), for tgtg-old as 11.78±1.63% (range: 9.88–13.31%) and for ctl-old as 0.61±0.17% (range: 0.34–0.79%). Differences between groups were tested corresponding to the staging of Aβ load in these groups (ctl-old against tgtg-young, tgtg-young against tg-old and tg-old against tgtg-old). The difference between all groups was highly significant (p<0.001). For all relative plaque load observations, no differences were found between right and left brain sides in all animals and between old and young control animals.

Plaque sizes considerably increased with age in hemizygous and homozygous animals (Figure 4B). After histogramming the individual plaque sizes for each transgenic group, and estimating kernel density functions, we could calculate that the differences between plaque size distributions of all transgenic study groups were highly significant (p<0.001). While relative plaque areas and size compositions were significantly different between the old study groups, the size composition of plaques in neocortex at old ages appeared to have a similarity independent of genotype.

### Cerebellum as reference region

Willuweit et al. reported that the cerebellum seems to be free of plaques in this animal model [31]. For our studies, we used the cerebellum as a reference region in PET, biodistribution and autoradiography. Therefore, we applied the histological quantification of Aβ plaques to the cerebellum, also, to analyze whether it qualifies as a reference region for imaging purposes in this animal model. For this, the same large dataset as above was used for the Thioflavin S (599 observations) and Aβ40/42 antibody (148 observations) modalities.

The Aβ42 antibody was providing for the fluorescent channel with the highest signal-to-noise ratio and was therefore the most reliable signal for the analysis of a potentially target-free region. The quantification results with the Aβ42 antibody were always lower than 0.09% compared to neocortex of tgtg-young animals which was larger than 3.09%. The average cerebellar binding of the Aβ42 antibody per group was 0.02±0.01% (range: 0.01–0.03) for tg-old, 0.01±0.00% (range: 0.01–0.02) for tgtg-young, 0.04±0.03% (range: 0.01–0.09) for tgtg-old and 0.02±0.01% (range: 0.01–0.02) for ctl-old. No significant differences could be seen between the groups: ctl-old to tgtg-young (p = 0.59), tgtg-young to tg-old (p = 0.25) and tgtg-young to tgtg-old (p = 0.17).

Thioflavin S showed unspecific binding behavior in tissues without Aβ deposits (Figure 2D) as described above. Nevertheless, the highest unspecific results in cerebellum were far below the lowest specific results in neocortex (0.82 vs 3.53%). The average cerebellar binding of Thioflavin S per group was 0.36±0.05% (range: 0.32–0.44) for tg-old, 0.25±0.06% (range: 0.16–0.33) for tgtg-young, 0.30±0.04% (range: 0.25–0.36) for tgtg-old and 0.66±0.13% (range: 0.45–0.82) for ctl-old. Differences between groups were significant, here, but it was the control groups that showed slightly higher binding than the transgenic animals.

These results for the cerebellum quantitatively confirmed that this region can be used as a reference region.

### Aβ40 and Aβ42 protein levels (ELISA)

Detailed differential Aβ protein analyses were performed in this animal model, previously, and tight correlations with relative Aβ plaque load were shown [31].

To further validate our PET imaging results and to understand how our in vitro tracer binding results with human and mouse brain homogenates (see below) relate to Aβ protein levels, brain tissue of 14 animals that received a PET scan, brain tissue of 8 animals and three samples of post-mortem human brain tissue for radioligand binding assay were biochemically quantified for human Aβ_x–40_ and Aβ_x–42_ protein (Table 1). Differential extraction procedures were applied in order to determine the levels of either soluble or insoluble forms of Aβ species for all samples.

In general, our results confirm the previous report [31]. Detailed individual results of ELISA analyses are shown in Table 3 and Table 4 for the corresponding experiments and are described there. The individual results and correlation of insoluble Aβ protein levels to results from PET imaging, autoradiography and histological plaque quantification are shown in Figure S10.

**Table 3:** In vitro binding potential and Aβ40/42 protein levels.

study group	soluble protein		insoluble protein		binding potential
	Aβ_x–40_	Aβ_x–42_	Aβ_x–40_	Aβ_x–42_	
**huCTL**	0.7	1	15.9	23.1	0
**huAD-0**	0.8	1.6	15.8	121.9	0
**huAD-C**	5.8	28.5	120.5	449.9	38.2
**tg-old**	517.8	186.2	169915.9	145010.5	11.9
**tgtg-young**	450.5	304.8	172152.3	178821.1	14.3
**tgtg-old**	912.4	485.4	657818.2	572182.5	69.2

**Figure 5:**
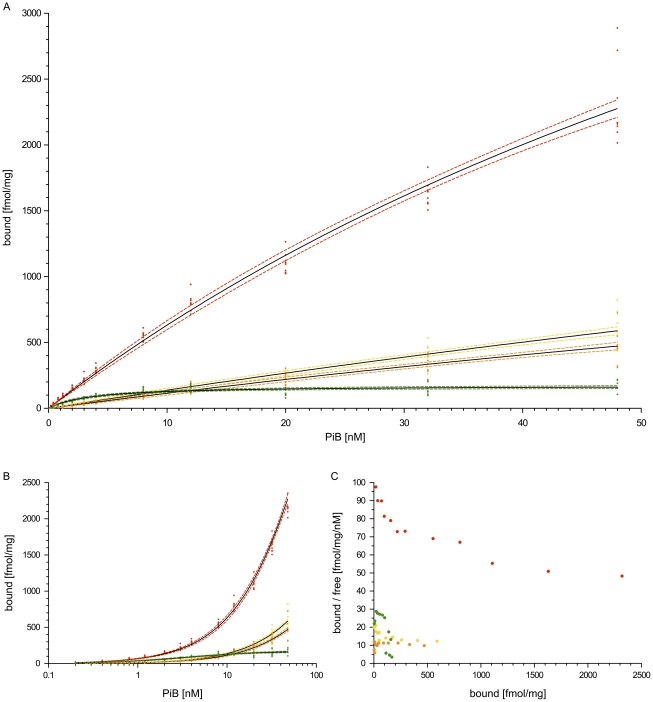
In vitro binding assay with [^3^H]PiB. Specific [^3^H]PiB binding to mouse and human brain homogenates and nonlinear modeling of data. (**A**) Binding isotherms for [^3^H]PiB with transgenic mouse brain tissues and human AD tissue containing Aβ deposits. Solid black curves show nonlinear fits with a single site model. Dashed lines describe 95% confidence bands around the fit. Resulting in vitro binding potential (BP) values and Aβ protein levels of these tissues are described in Table 3. (**B**) Semilogarithmic representation of the specific binding data as seen in panel (A) to delineate possible infliction points. (**C**) Scatchard graphs showing the same data as panel (A). Each data point is derived from the mean value of the original data octuples. Data show representative samples from tgtg-old (*red*), tgtg-young (*yellow*) and tg-old (*orange*) transgenic study group and severely affected human AD (huAD-C) tissue (*green*).

In vitro binding potential (BP) as yielded with [^3^H]PiB radioligand saturation binding assay and corresponding soluble and insoluble Aβ_x–40_ and Aβ_x–42_ protein fractions (picogram protein per milligram wet tissue) for the same human and mouse tissue samples. Binding curves of the severe human AD and all transgenic mouse brain samples are shown in Figure 5.

**Table 4:** Multi-modal combined experiment with [^3^H]PiB/[^11^C]PiB cocktail.

modality			AD1*	AD2	CTL1*	CTL2
PET [BP_ND_ neocortex]			0.06	0.06	−0.06	−0.07
Biodistribution [^11^C]PiB [region-to-cerebellum ratio]		olfactory system	1.00	1.07	1.05	0.98
		telencephalon	1.24	1.24	0.92	0.92
		diencephalon and midbrain	1.26	1.47	1.22	1.10
Autoradiography [neocortex-to-cerebellum ratio]		[^11^C]PiB	1.88	2.1	0.72	0.95
		[^3^H]PiB	1.26	1.24	0.90	0.97
Histology [% plaque area neocortex]		Thioflavin S	4.00	4.10	0.54	0.41
		Aβ_x–40_	3.63	3.57		
		Aβ_x–42_	5.88	6.14	0.02	0.01
Aβ protein levels (forebrain) [pg protein/mg tissue wet weight]	soluble	Aβ_x–40_	272.0	212.1		
		Aβ_x–42_	267.3	191.3		
	insoluble	Aβ_x–40_	91522.8	117849.6		
		Aβ_x–42_	134335.4	164156.0		

**Figure 6:**
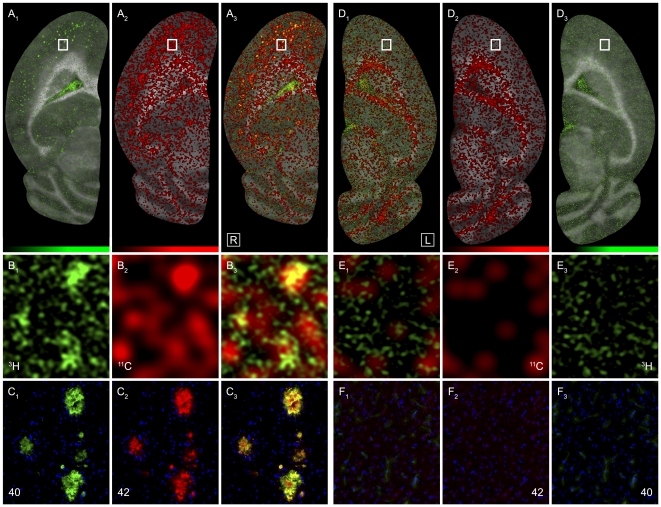
Multi-modal combined experiment. Single multi-modal in vivo/ex vivo combination experiment with 4 animals from the young study groups (2 tgtg-young and 2 ctl-young) showing the whole spectrum of results on an individual level. After a bolus injection of a [^11^C]PiB/[^3^H]PiB cocktail, the animals passed a 30 min CT/PET scan, were then killed for immediate [^11^C]PiB regional brain biodistribution and dual-label digital autoradiography. Brain halves used for biodistribution were analyzed for Aβ protein levels. The other brain halves were stained with Thioflavin S and anti-Aβ40/42 and used for histological plaque quantification. *Columns* (**1**) to (**3**): 9 month old female homozygous APP/PS1 mouse (“AD1”) and *Column* (**4**) to (**6**): 9 month old female C57BL/6J control mouse (“CTL1”), presented in a mirror fashion. Ex vivo [^11^C]PiB (*red*)/[^3^H]PiB (*green*) dual-label digital autoradiographs with underlying optical scans of horizontal 12 µm half brain sections of AD1 (right brain) (**A** and **B**) and CTL1 (left brain) (**D** and **E**) (marked with asterisk (*) in Table 3) and corresponding magnified views of double immunofluorescence stains for Aβ40 (*green*) and Aβ42 (*red*) of neighboring sections for the same region (**C** and **F**). All four modalities are shown individually (*outer two columns*) and co-localized (*central columns*). Limits of green and red color look-up-tables represent minimum and maximum of measured signal. The analytical results of all experiments are shown in Table 3 below this figure.

An all-in-one experiment was performed for four animals of the young study groups (2 tgtg-young (AD1 and AD2) and 2 ctl-young (CTL1 and CTL2)) to retrieve a large spectrum of multi-modal information from a single animal. Asterisk (*) marks animals that are shown in Figure 6.

### In vitro [^3^H]PiB binding assay

In vitro tracer binding to brain homogenates provides a sensitive reference to the other experimental modalities [33]. Therefore, seven samples of brain tissue were used for assessing in vitro [^3^H]PiB binding to mouse brain tissue and postmortem human brain tissue at distinct disease stages (mouse: tg-old, tgtg-young, tgtg-old; human: Cerad-C/Braak V and Cerad-0/Braak II). Binding to postmortem human brain tissue was performed to provide a reference for the results from mouse brain tissue. A binding assay to synthetic Aβ_1-40_ fibrils was also performed and used as positive control (Figure S8). The huAD-C (Cerad-C/Braak V) tissue sample was retested twice (Figure S9). All brain tissues used for in vitro binding were also analyzed for Aβ protein levels with ELISA (Table 3).

Some tissues were lacking sufficient [^3^H]PiB binding saturation behavior (Figure 5). Hence, in vitro binding potential (BP) was chosen as the target value for tissue comparisons after global nonlinear regression with the single binding site model to total and nonspecific binding data (Table 3). As K_d_ and B_max_ are correlated, BP can be measured very accurately either as ratio from the estimates, as done here, or from the initial slopes to the specific binding curves [49] even though independent estimates for K_d_ and B_max_ may not reasonably be possible.

#### Human brain

The severely affected human AD tissue (huAD-C) homogenate provided an estimate for BP of 38.2±4.7 (K_d_ = 5.2±0.9 nM, B_max_ = 200±12 fmol/mg) for [^3^H]PiB. Figure 5 shows how the huAD-C tissue homogenate reached saturation binding at comparably low concentrations of the tracer (around 8 nM).

While the huAD-C sample yielded binding values corresponding to previous reports for severely affected human AD brain tissue [8], [50], [51], the total binding data of the mildly affected human AD sample (huAD-0) and the human control brain (huCTL) was showing no difference to nonspecific binding data (data not shown) indicating that a binding component which could compete with 3 µM PiB was not present (BP estimates close to 0). This corresponded to the Aβ protein quantification results (Table 3) in spite of repeated positive neuropathological staging according to BrainNet Europe standards.

In ELISA, human Aβ-free matched control tissue was included to provide reference values. The huAD-0 sample showed soluble and insoluble Aβ_x–40_ levels comparable to control tissue, while Aβ_x–42_ levels were increased and at about one third of the huAD-C sample for the insoluble fraction. The Aβ_x–42_ levels of huAD-C were more than fourfold to the Aβ_x–40_ levels of the same sample.

#### Mouse brain

The initial steepness of the binding curves for mouse brain tissue homogenates was comparable to and even higher (tgtg-old) than for the severely affected human AD tissue (Figure 5). This binding behavior at low tracer concentrations is considered a prerequisite for successful PET imaging and confirms our positive PET imaging outcomes described above.

BP in the transgenic mouse brain tissues were estimated to 11.9±0.9 (tg-old), 14.3±0.9 (tgtg-young) and 69.2±2.7 (tgtg-old). The BP of homozygous old mouse brain tissue was clearly above the value for severely affected human AD tissue while the tg-old and tgtg-young samples were at about one third of the result for huAD-C. The total binding data of mouse control brain (msCTL), like the huAD-0 and huCTL samples was not different to nonspecific binding (data not shown) indicating that a binding component which could compete with 3 µM PiB was not present (BP estimates close to 0).

Independent estimates for K_d_ and B_max_ of the high-affinity component were yielded for the tgtg-old tissue as it reached a sufficient degree of tracer saturation binding. Fitting these data to the two site binding model revealed a K_d_ of 5.7±4.1 nM and a B_max_ of 289.3±123.9 fmol/mg while the huAD-C data provided a K_d_ of 3.4±1.4 nM and a B_max_ of 156.7±7.3 fmol/mg as estimates for the same fit.

Scatchard graphs corresponding to the specific binding curves are displayed additionally in Figure 5, together with a semilogarithmic representation of the specific binding curves. The semilogarithmic plot shows the absence of infliction points in the data of tg-old and tgtg-young which would be necessary for the distinction of different binding sites and for the ability to fit one or two lines (one- or two-site model) to the Scatchard data [52].

In accordance to previous reports [33], the Aβ levels of mouse brain tissue were higher than those found in human AD brain by a factor of around 1000 (Table 3). Furthermore, the mouse Aβ levels also seem to indicate a correspondence to measured BP values.

### Combined multi-modal experiment

To bring together a large set of experimental modalities applied to an individual animal, and to cross-validate and address their relationship within a single animal, a combined experiment was performed (Figure 6 and Table 4) consisting of PET, regional brain biodistribution, dual-label digital autoradiography, histological Aβ plaque quantification with Thioflavin S and anti-Aβ40/42 and Aβ40/42 ELISA.

Four animals from the young study groups (2 tgtg-young, 2 ctl-young) were given a bolus cocktail of [^11^C]PiB/[^3^H]PiB in the PET scanner and their brain tissue processed immediately after PET imaging. The individual results for all four animals are shown in Table 4. The good correspondence of ex vivo [^11^C]PiB and ex vivo [^3^H]PiB autoradiography together with double anti-Aβ40/42 fluorescent stains of a neighboring section are shown in Figure 6.

This combination experiment showed how excellent the results from different experimental modalities correlate on the level of individual animals and how activity uptake in PET is real tracer uptake. Also, these individual results confirmed that young homozygous animals could clearly be distinguished from control animals in all modalities. Furthermore, it showed the consistency and robustness of the groupwise results described above on an individual analysis level.

### Relationship of in vivo PET to other experimental modalities

The study was designed to provide as many validation experiments to PET imaging in every animal as possible, in order to analyze the relationship of in vivo radioligand binding in PET to relative ex vivo tracer uptake in brain biodistribution and autoradiography, to histological Aβ plaque burden and to Aβ protein levels (Table 1).

Neocortex, i.e. complete cortex without hippocampus, was used as the primary target region. It was defined in the same way on horizontal PET slices (Figure S4) and horizontal autoradiographical and histological sections. The brain region that was used for ELISA analysis was defined as previously reported [31] and hence contained telencephalon and the largest part of diencephalon and midbrain without the olfactory system. This presumably provided for slightly weaker correlations of Aβ protein levels with the other methods.

**Figure 7:**
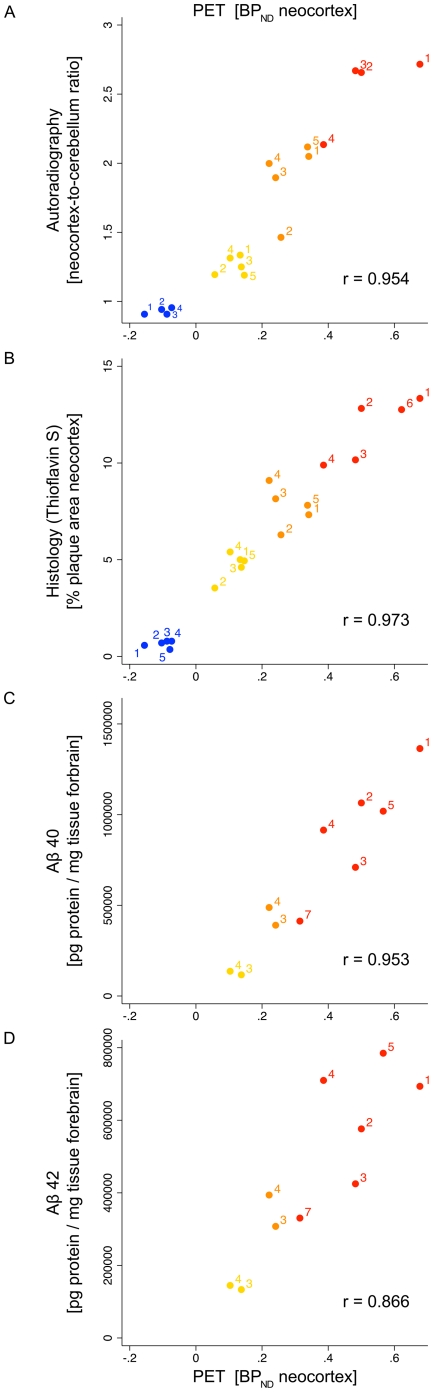
Relationship of in vivo PET to other experimental modalities. Association of in vivo [^11^C]PiB binding potential in mouse neocortex with relative neocortical [^3^H]PiB uptake in autoradiography (**A**), with relative neocortical Aβ plaque burden as stained by Thioflavin S (**B**) and with insoluble Aβ40 and Aβ42 protein levels in forebrain (**C** and **D**). Data across the modalities was acquired from tissue of the same animals (as shown in Table 1). Individual animals are identified by their unique number code within their study group. The coloring of study groups in the scatter plots shows how each group is fully separated from each other. Color code: tg-old (*orange*), tgtg-young (*yellow*), tgtg-old (*red*) and ctl-old (*blue*). Pairwise correlation coefficients (r) for each pair of modalities are noted in each scatter plot. Histological quantification with Thioflavin S is used representatively for all histological quantification results because of its tight correlation with anti-Aβ40/42 as described in Figure S7. Here, the animals presented in Figures 1 and 2 are coded with #5 (tg-old), #5 (tgtg-young), #1 (tg-old) and #1 (ctl-old). The full scatter matrix for the cross-validation of experimental results is shown in Figure S10.

Figure 7 summarizes these relationships for the animals of the three transgenic study groups and the old control group. In general, [^11^C]PiB binding in PET was correlating strongly with ex vivo tracer uptake and in vitro Aβ load. In addition, each study group was clearly separate from each other in all experimental modalities (shown by different color for each study group). The summary of data demonstrates the robustness of the small-animal PET results and their consistency with the validation experiments. A complete overview of the cross-validation approach is shown in Figure S10.

## Discussion

The need for more specific imaging markers of Aβ, tau and alpha-synuclein [53] requires robust and feasible translational imaging tools to enable the evaluation and ranking of new tracers. Previous PET imaging studies with [^11^C]PiB in different AD mouse models were not successful, despite high Aβ plaque loads [33], [38], [39] and the only successful study relied on very high specific activities of the tracer [41]. This led researchers to principally question the feasibility and potential of this imaging method for translational AD research [32], [33], [40], [54].

For these reasons, we addressed the development of a feasible, reproducible and robust preclinical Aβ plaque PET imaging setup in transgenic AD mice that reliably detects a specific signal in animals young enough to allow for longitudinal follow-up studies. We were able to show that the measured uptake of [^11^C]PiB with PET in individual transgenic animals at different disease stages was robust and strongly correlated with several independent experimental methods in the same animals.

The old hemizygous AD group was selected to correspond to animal ages used in previous imaging studies [33], [38], [41]. In general, the results of this group tended to be relatively close to the young homozygous animals. These results show the value of the homozygous animals of this APP/PS1 mouse model for imaging: the reliable specific PET signal in young animals in combination with a virtually normal life span and low premature death rate of homozygous mice allows for sufficiently long follow-up studies.

The PET results in control animals are notable as they indicate a volume of distribution ratio <1 (cerebellum as reference) and hence a larger volume of distribution for the cerebellum than for neocortex. This finding is consistent with Maeda et al. [41] and is probably related to white matter binding of the tracer in the cerebellum in contrast to the target region which does not contain white matter. It is further supported by our regional brain biodistribution results which also yielded ratios <1 for telencephalon-to-cerebellum ratios of injected tracer doses normalized to tissue weight.

The considerable amount of unspecific PiB retention in tissues outside of the brain (like the salivary and harderian glands) is likely to be model-independent. Previous studies [33], [38], [41] do not provide information on whether the applied PET technologies have been able to resolve the uptake in extracerebral regions neighboring the olfactory bulb and the frontal cortex. In our study, we have identified these issues as a potential error source and it highlights the importance of precise PET image co-registration to MRI such that volumes-of-interest can be defined reliably. In our experience, manual image co-registration of well pre-processed small-animal data yields excellent and very reliable results. A similar manual method has been reported by Pfluger et al. for human MRI-SPECT data [55].

In a different APP/PS1 model, Klunk et al. detected an uptake of [^11^C]PiB of 100–120% in the entire cerebrum relative to PS1 mice [33]. Their results were not statistically significant, which may have been due to the small sample size (1 transgenic versus 1 control animal per age group) and the global VOI-based approach employed (large VOI encompassing the entire brain, no reference region). In another study, Toyama et al. included a reference tissue-based analysis in their study with six Tg2576 mice at a mean age of 22 months, using the cerebellum as a reference region [38]. Although they calculated significantly higher binding ratios in the transgenic mice, Toyama et al. concluded that their study could not prove specific binding of [^11^C]PiB to Aβ plaques due to the overall small difference in absolute tracer uptake between transgenic and control animals which may have been due to the presence of Aβ plaques in the cerebellum of this animal model [24]. The sole report on successful in vivo [^11^C]PiB imaging with PET in a single transgenic mouse model (APP23) claimed extraordinarily high specific activities of their PiB preparation (max. 291 GBq/µmol) to be required for imaging of Aβ plaques in their animals [41]. Specific activities of this magnitude are not obtainable at most PET centers which may explain why these results have not been reproduced by others. Furthermore, the proportionality between tracer uptake and Aβ plaque load as derived from a small-animal PET study may not be transferable to humans, if 10 to 20-fold higher specific activities are applied in the animal model. Another relevant aspect of the study of Maeda et al. is that reasonable tracer uptake has been found in animals >21 months of age, despite the high specific activity preparations. This further limits the applicability of this imaging protocol for follow-up studies as the average life span of APP23 mice is around two years [27], [56].

In our study, significant tracer uptake in regions with Aβ plaques was demonstrated in transgenic mice as young as 9 months injected with 28.9±7.93 MBq of [^11^C]PiB in a specific activity of 11 GBq/µmol. Hence, our specific activities were about 20-fold lower and better comparable to that routinely applied in studies of AD patients (range: 11.1–14.8 GBq/µmol). An even higher molar amount of [^3^H]PiB (2.5 nmol) was used for the ex vivo autoradiography studies compared to our in vivo PET studies (1.6 nmol of PiB) which both demonstrated specific binding of the tracer to Aβ plaques. Several factors may be responsible for this discrepancy of our findings compared to previous work. The choice of animal model may be a key explanation.

In contrast to the abundance of available transgenic AD models with high content of cortical Aβ plaques [57], good preclinical models for imaging Aβ deposits have still been lacking [32], [40]. However, various ex vivo analyses with Aβ ligands in AD mice [31], [33], [58], [59] and in vivo fluorescent labeling [36], [60] would suggest that the in vivo measurement of Aβ plaque load in mice with PET should be possible. In humans, negative PiB imaging in severely amyloid-positive patients with the arctic APP mutation has been observed [61] and may indicate a parallel phenomenon to negative PiB-PET results in animal models. In which way different Aβ isoform patterns [62] and their degree of fibrillarity [63] contribute to PET imaging results remains to be examined.

A number of previously reported favorable characteristics of the APP/PS1 mouse model employed in our study [31] probably contribute to the observed positive findings for several reasons. The potential advantages are: a) an early-onset and rapid progression of plaque load, b) plaques showing similar morphology to those in human AD, c) low inter-animal variability and no gender effects (in contrast to other transgenic animal models which show high variability of Aβ plaque expression [27]), d) co-inherited transgenes and a C57BL/6 background leading to good breeding capabilities of a homozygous line and a low rate of premature death of hemizygous and homozygous mice up to normal old age [31]. The homozygous animals show earlier onset and more rapid progression of Aβ plaque deposition compared to hemizygous animals and are therefore good candidates for at least one and a half years of longitudinal imaging.

While some other models develop Aβ deposits in the cerebellum over time [24], [30], we could show that the cerebellum of our model stays free until old age and can therefore be used for reference tissue approaches. This is an important feasibility advantage as alternative methods for analysis require arterial input information and calibration to injected dose both of which remain methodological challenges.

The plaque quantification results in our study differ somewhat from what has been reported, previously [31]. Willuweit et al. measured relative plaque burden in 19 to 20 months old transgenic mice of 10.5% (hemizygous) and 35.2% (homozygous). Here, the measured relative plaque burden in even older animals were lower and the results lay closer together (around 5% and 12%). There may be several methodological reasons for this. Firstly, we have used frozen brain material for this study while Willuweit et al. took paraffin sections. Secondly, we have used other primary antibodies for Aβ40 and Aβ42 detection with a different staining protocol. Thirdly, the parameters for the automated plaque detection algorithm needed to be adjusted to histological material, stain and exposure times.

Thioflavin S is an easy to use staining agent and was applied for histological Aβ plaque quantification, before [64]. Here, we applied our semi-automatic imaging algorithm [31] to Thioflavin S stained sections for the first time and validated this approach in relation to Aβ40 and Aβ42 antibodies.

The dual-label autoradiographs (Figure 6) show different nonspecific binding of [^11^C]PiB and [^3^H]PiB to mouse brain tissue. While [^11^C]PiB was also taken up by white matter (seen best in panel D_2_), nonspecific [^3^H]PiB binding was mostly observed in vessels (seen best in the choroid plexus). Both versions of PiB (i.e. labeled with either ^3^H or ^11^C) had a similar specificity to Aβ plaques. The specific activities of both versions of PiB were the same as they were applied in a cocktail bolus. However, the measurement of uptake ratios relative to cerebellum in autoradiography is less reliable with [^11^C]PiB, as slight deviations in thickness within a section has direct influence on the ratios. This effect is negligible when using [^3^H]PiB ratios due to less energy of tritium.

In vitro PiB binding was first studied in transgenic mice by Klunk et al. who found only a very small high-affinity component for [^3^H]PiB in very old hemizygous APP/PS1 animals (BP = 78) [33] using classical Scatchard analysis. The authors reasoned that the low concentration of high-affinity binding sites compared to humans (BP = 636) might be the reason for unsuccessful PET imaging in mice.

Our results agree with some aspects of what has been reported by Klunk et al. (e.g. different binding kinetics for rodent AD tissue and correlation of B_max_ with insoluble Aβ), while it deviates in other points (e.g. existence of high-affinity binding component and comparable BPs). Using a state-of-the-art global nonlinear regression approach for analyzing total and nonspecific binding data for a larger range of tracer concentrations, we revealed a higher BP ( = 12) in young hemizygous animals relative to severely affected human AD tissue ( = 38). Furthermore, the BP ( = 69) of our old homozygous mouse brain sample was nearly double compared to human.

The slow tracer saturation in AD mouse brain indicates a different binding behavior to rodent Aβ plaques in the presence of considerably higher levels of Aβ in mouse than in human [54]. It may be difficult to identify a high-affinity component in hemizygous old mouse brain tissue. Homozygous old mouse brain tissue, however, provides a definite high-affinity binding component comparable to human AD tissue.

A direct correlation between B_max_ and insoluble Aβ content was reported by Klunk et al.. In our study, an association between BP and insoluble Aβ in the transgenic mice was observed. As one may assume that the affinity of [^3^H]PiB to the binding sites of various APP/PS1 mouse brain tissues of the same genetic strain is similar and that, hence, BP is mostly related to B_max_ [49] these observations seem to be similar.

In summary, we have provided a cross-validated study for feasible small-animal PET imaging of Aβ plaque deposition with [^11^C]PiB in an APP/PS1 mouse model of Alzheimer's disease. In vivo PET imaging results of three different transgenic mouse study groups and matched control groups were validated with ex vivo and in vitro methods. The transgenic study groups represented different disease stages according to their Aβ pathology and could well be distinguished with PET. Group results were consistent in all experimental modalities and individual results correlated tightly. The reported PET imaging protocol uses readily achievable levels of specific activity of the tracer and grants successful high-contrast imaging down to ages of at least nine months. This provides the opportunity for at least one and a half years of longitudinal studies and, hence, truly translational Aβ plaque imaging of Alzheimer's disease in a preclinical model. The established imaging setup and multi-modal cross-validation protocol are applied to our tracer development program for ranking and successful evaluation of new imaging markers for Aβ [18], [19].

## Materials and Methods

### Animals

The experiments were carried out with the approval of the institutional animal care committee (Regierung von Oberbayern, Munich, Germany) and in accordance with the German Animal Welfare Act (Deutsches Tierschutzgesetz). Animal husbandry followed the regulations of European Union (EU) guideline No. 86/609.

All experiments were performed in hemizygous (tg) and homozygous (tgtg) APP/PS1 mice (B6;CB-Tg(Thy1-PSEN1*M146V/Thy1-APP*swe)-10Arte) (TaconicArtemis GmbH, Cologne, Germany) on a congenic C57BL/6J genetic background and commercially available age- and gender-matched controls (Harlan-Winkelmann, Borchen, Germany and Janvier, Le Genest-St-Isle, France). The transgenic mouse model has been characterized regarding onset, progression, distribution and extent of Aβ plaque deposition as well as behavioral features [31].

The animals were kept under temperature-controlled environmental conditions (18–20°C, 50–60% relative humidity) on a 12∶12 light-dark cycle (light from 6 am to 6 pm) and fed a standard diet (Altromin 1326 mouse pellets, Altromin, Lage, Germany) with free access to food and potable water until the start of the experiments and after (no fasting). They were group-housed (maximum of 5 individuals per group) in individually ventilated type III cages (Ehret, Emmendingen, Germany) with dust-reduced wood shavings as bedding. All animals underwent a minimum of 10 days acclimatization period.

### Study design

Altogether 70 animals in five study groups were used in this study such that groupwise and pairwise comparisons are possible. Group age definition of animals was chosen to be “young” (9 months) and “old” (21 and 23 months). Three transgenic study groups of hemizygous (tg-old) and homozygous (tgtg-young, tgtg-old) animals were included to provide comparability with previous reports and also to show how the imaging outcome can be improved by using homozygous animals. In previous pilot studies, young animals from seven to ten months were tested (unpublished data). These preliminary results revealed reliable and satisfactory Aβ plaque detection and visualization with PET and we found an age of nine months to be a feasible age definition for the young study group. The two control study groups (ctl-young and ctl-old) were designed to match gender and age and to additionally control for any differences among the controls regarding gender (female and male subgroups). Regarding body weight, female transgenic animals tend to weigh less than female controls and female controls weigh less than male controls. To our experience the unavoidable differences in weight have no detectable influence on the results presented, here.

Table 1 shows a detailed description of the study collective and the combination of experiments performed for each subgroup.

### Postmortem human brain tissue

Three samples of deep frozen human brain tissue were provided by Neurobiobank Munich upon request to BrainNet Europe (www.brainnet-europe.org) after approval of the ethics committee at Technische Universität München. Neuropathological diagnostics were performed according to BrainNet Europe standards.

All samples were taken from temporal cortex gray matter of three female donors who died at an age of 79 to 85. Significantly different amyloid-beta plaque load (as confirmed with 4G8 antibody stain) was a major selection criterium. Hence, one severe AD brain staged as Cerad-C, Braak V (“huAD-C”), one mild AD brain (“huAD-0”) staged as Cerad-0, Braak II and one age- and gender-matched control brain (“huCTL”) staged as Cerad-0, Braak I (4G8-negative) were chosen.

### Radiosynthesis

2-(4′-amino-phenyl)-6-OH-benzothiazole (6-OH-BTA-0) and 2-(4′-*N*-methylamino-phenyl)-6-OH-benzothiazole (6-OH-BTA-1) were purchased from ABX Biochemicals, Radeberg, Germany. Other reagents and solvents were purchased from Sigma-Aldrich. Chromatography columns were from CS-Chromatographie (Langerwehe, Germany). HPLC pumps and UV detectors were from Sykam (Fuerstenfeldbruck, Germany).

Cyclotron-produced [^11^C]CO_2_ was converted to [^11^C]CH_3_I by the catalytic gas-phase iodination reaction via [^11^C]CH_4_ (GE MeI MicroLab) and converted [^11^C]CH_3_OTf by distillation through a column of AgOTf impregnated on α-alumina. Subsequent radiolabeling and purification was carried out in a fully automated synthesizer from Scintomics (Fuerstenfeldbruck, Germany).

10 µmol of the primary amine 2-(4′-amino-phenyl)-6-OH-benzothiazole (6-OH-BTA-0) was dissolved in anhydrous acetone (250 µl). The vial was sealed, flushed with and maintained under argon. The [^11^C]CH_3_OTf produced, swept with a He-flow at 50 ml/min, was trapped in the reaction vial. The reaction vial was warmed to 65°C over 30 s and kept at this temperature for 2 min. Thereafter the reaction mixture was diluted with 1 ml of MeCN: 0.1 M ammonium formate (27.5∶72.5, ^V^/_V_), loaded into a 2 ml injection loop and transferred onto a μ-Bondapak C_18_ column (10 µm particle size; ID of 8 mm; length of 300 mm; CS-Chromatographie). The column was eluted with a mobile phase consisting of MeCN: 0.1 M ammonium formate (50∶50, ^V^/_V_) at a flow rate of 4 ml/min. In-line HPLC detectors included a UV detector (Sykam) set at 254 nm and a γ-ray detector (Bioscan Flow-Count fitted with a PIN detector).

For animal experiments, the fraction containing the product was collected in a rotary evaporation flask containing 1 ml of 1% HCl in EtOH and evaporated to dryness under reduced pressure. The product was dissolved in 1 to 2 ml of phosphate buffered saline (PBS). The pH of the final solution was between 7 and 8.

Analytical HPLC system 1 was a Nucleosil 100 5 µm CN 4.6×250 mm reverse phase column (CS-Chromatographie) eluted with acetonitrile/0.1 M ammonium formate (55∶45, ^V^/_V_) mobile phase mixture. The flow rate was 1.0 ml/min. HPLC system 2 was a Nucleosil 100 5 µm C_18_ 4.6×250 mm reverse phase column (CS-Chromatographie) eluted with acetonitrile/0.1 M ammonium formate (55∶45, ^V^/_V_). The flow rate was 1.0 ml/min. The capacity constant, *k*′ (*k*′ = t_R_−t_0_/t_0_) for *N*-[^11^C-methyl]-6-OH-BTA-1 on HPLC system 1 was 3.2 and for system 2 2.1. The ^11^C-labeled product co-eluted with an authentic standard of 6-OH-BTA-1.

Radiochemical and chemical purities were >98.5% as determined by analytical HPLC. The radiochemical yield averaged 35% at the end of synthesis (EOS) based on [^11^C]CH_3_OTf and the specific activity averaged 76.7 GBq/ µmol at EOS.

Usually, two animals sequentially underwent PET scans with the tracer as prepared from a single synthesis. In order to inject an identical chemical amount of substance for the two scans, an amount of authentic standard *N*-methyl-6-OH-BTA-1 was added to the first injectate. This resulted in a specific activity of the preparations in the range 300–400 mCi/µmol (11.1–14.8 GBq/µmol).

### Anesthesia

Inhalation anesthesia was used for PET scans, metabolites and biodistribution experiments. Anesthesia was begun 15 min ahead of experimental procedures by placing the animal in a cage ventilated with isoflurane (3%) and oxygen (3.5 l/min) with a pre-calibrated vaporizer. During the experiments, anesthesia was maintained by 0.6% to 2% isoflurane and 3.5 l/min oxygen via a nose cone, depending on length of scan such that the respiratory rate stayed at 80–100/min. Body temperature was held at 37°C with a temperature-controlled heating pad.

Peritoneal antagonisable triple anesthesia with medetomidine, midazolam and fentanyl (MMF) was used for all animals during the MR scan.

Whenever anesthetized the eyes of each animal were protected with dexpanthenol eye ointment.

### Substance administration

All injections were performed under isoflurane inhalation anesthesia. An application catheter system for reliable intravenous access to the lateral tail veins was prepared using 30 gauge needles, polyethylene tubing (0.28 mm inner diameter), superglue and 1 ml syringes. To achieve reliable and long-term access, an elastic hollow vessel-loop was used as a tourniquet for catheter placement. The catheter and syringe were initially filled with isotonic sodium chloride solution. The functional catheter was stabilized at the injection site with superglue. For dual-tracer PET scans, a catheter system was placed in each of the two tail veins for independent application of the radiotracers.

### Small-animal PET with [^11^C]PiB

#### General PET scanning procedure

Most of the small-animal PET data was acquired with a microPET FOCUS F120 scanner (Siemens Medical Solutions, Malvern, USA) [65]. In the combined multi-modal experiment PET data for the four animals was acquired with a docked Siemens Inveon PET/CT system (Siemens Medical Solutions, Knoxville, USA) [21], [66], [67].

After induction of anesthesia and placement of the catheter systems, the animals were placed with their heads in the center of the field of view and were fixed in the scanner in prone head first position (HFP). At the beginning of the PET scanning procedure, a 9 min transmission scan (^68^Ge rod source, Focus F120) or CT scan (Inveon) was performed in all animals.

[^11^C]PiB was given via the catheter system intravenously in a slow bolus, followed by flushing with isotonic saline solution such that the total applied volume was 0.22±0.06 ml. The amount of injected activity was controlled real-time with registered prompts such that they ranged between 150000 to 200000 at the end of [^11^C]PiB application, ensuring a dead time <5% at 30 min p.i.. The radioactivity in the syringe was measured immediately before and after injection with a Capintec CRC 15R (Capintec Inc, NJ, USA) dose calibrator. The time between measurements was 2 to 3 min.

Dynamic data acquisition was performed in 3D listmode for 30 or 60 min starting immediately with injection of the tracer. The emission data were normalized and corrected for decay and dead time. The resulting sinograms were reconstructed with FBP (filtered back-projection using a ramp filter with a cut-off at the Nyquist frequency) into 2, 3, 6, 12, 60 and 120 frames of equal length used for motion correction, ratio measurements and image production and 52 frames (24×10 s, 12×30 s, 10×120 s, 6×300 s) and 162 frames (120×1 s, 24×10 s, 8×30 s, 10×300 s) for time-activity-curve (TAC) generation. The image volume consisted of 128×128×95 voxels, with a size of 0.866×0.866×0.796 mm^3^ per voxel for the Focus F120 scanner and 128×128×159 voxels, with a size of 0.776×0.776×0.796 mm^3^ per voxel for the Inveon scanner. Test-retest studies (1 week apart) with five transgenic animals showed robustness of PET results for these measurements in mouse brain.

#### Sequential dual-tracer PET scans with [^11^C]PiB and [^18^F]FDG

To gain additional information for manual PET and MRI co-registration and to verify that the anatomical localization of unspecific [^11^C]PiB uptake in vivo is extracerebral, about one fifth of the animals in our PET imaging protocol were scanned sequentially with [^11^C]PiB and [^18^F]FDG (30 or 60 min) without being moved in the PET scanner.

#### CNS removal during PET

The preparation of animals and scanning setup were identical to the general PET protocol described above. At 30 min p.i., the scan was interrupted and the animal was guillotined. The complete brain (including the olfactory bulb) was taken out of the skull such that cerebral and cranial anatomical structures remained intact. The remaining head and the isolated brain were placed separately in the field-of-view of the scanner for another 30 min (ex vivo [^11^C]PiB PET).

### Mouse brain MRI

MR scans were performed the same day as PET, immediately following the application of [^3^H]PiB for later autoradiographic analyses. Anesthesia was switched to peritoneal MMF and the animal was transferred to the MR scanner. Animals were placed prone head first (HFP) in the MRI scanner (Philips Achieva 1.5 T clinical MRI system). Mouse CNS MRI was performed using a 23 mm microscopy coil fixed horizontally over the head of the animal. A Philips T1-weighted 3D turbo gradient echo sequence with an inversion pre-pulse was used: flip angle 8°, TR 13 ms (shortest), TE 4.3 ms (shortest), TI 860 msec, FOV 64 mm, pixel matrix 256^2^ reconstructed to 512^2^, section thickness 0.25 mm, interpolated to 0.125 mm. The scan time of the sequence is 46 min 11 sec.

### PET data analysis

All in vivo image data was processed and analyzed with PMOD 3.2 software package (Pmod Technologies, Zürich, Switzerland). All PET, MRI and CT image datasets were scaled to calibrated kBq/cc and saved in float format. Orientation of planes was conform to radiological human brain standard such that the Z-axis was perpendicular to horizontal sections. The median sagittal plane was co-registered to the median sagittal plate (no. 101) of Paxinos atlas [68]. Image origins were set to Bregma (0,0). All datasets were controlled for motion of the animal during the PET scan in image reconstructions with 60 and 120 frames of equal length, i.e. 60 s and 30 s per frame.

#### Image co-registration and quality control

To retrieve reliable small-animal PET results, accurate and standardized co-registration of PET to MRI or CT is essential. Deviations in the range of a single PET voxel may cause considerable differences (Figure 3 and Figure S2).

To create an in vivo correspondence to the Paxinos atlas space [68], sagittal atlas plate no. 101 was loaded into Pmod. The anterior-posterior and left-right axis of a high-resolution mouse head CT was aligned, the sagittal plane co-registered to the Paxinos plate and origins set to Bregma (0,0). The co-registered CT was cropped to a bounding box of 12×18×8 mm (x, y, z). Spatial correspondence to brain structures was verified with co-registered MRI datasets.

To provide a reliable basis for co-registration of PET datasets, all MRI datasets were co-registered to the Paxinos atlas space by creating an MRI template and using the automatic rigid matching functionality of Pmod with the normalized mutual information dissimilarity function for all individual MRI datasets.

To improve the manual co-registration of [^11^C]PiB scans, about one fifth of the animals also received [^18^F]FDG for 30 to 60 min immediately after the [^11^C]PiB scan without being moved to provide for identical transformation matrices of both scans. The advantage of [^18^F]FDG to delineate brain morphology was used for a more reliable co-registration of PiB scans (Figure S2).

A two-step matching process of PET data was used. A PET template of early tracer entrance (first 4 min) (Figure S3) was created for initial automatic rigid matching with the normalized mutual information dissimilarity function [69]. Automatic matching results were verified and corrected if necessary by applying the following quality control procedure: the PET template was color-coded with a red binary look-up-table (LUT) and the energy window set for the contour to delineate the brain. The PET study was color-coded with a green binary LUT and the energy window set accordingly. This quality control step was performed at various energy contour levels in all three planes of view and the co-registration corrected manually if necessary. This procedure was repeated with the co-registered MRI datasets as individual references. The pre-matched PET study was colored with a binary LUT and evaluated on all orthogonal slices using at least three different energy windows for the LUT contour.

#### Volumes-of-interest (VOI) definition

An MRI template in Paxinos atlas space was created from the individual co-registered MRI datasets of all transgenic animals of this study and, together with a high-resolution CT scan, used as the basis for VOI definition (Figure S4) according to Paxinos atlas [68] and the mouse brain atlas provided by the Allen Institute for Brain Science [70]. Paired brain structures were defined individually for right and left side and were also merged. The mouse brain cortex was defined in two subvolumes (neocortex and hippocampus). The following cerebral VOIs were defined (right and left sides of paired structures summed, volumes reported in brackets as mm^3^): whole brain (504.8), cerebellum (48.7), neocortex (101.3), hippocampus (39.4), thalamus (15.0) and olfactory bulb (14.6). Additionally, three extracerebral VOIs were defined for the evaluation of unspecific tracer retention: nasal sinus (39.9), harderian glands (32.3) and eyebulbs (14.4).

#### Quantification of dynamic PET data

To assess varying PiB retention of individual animals within the study collective and to verify the consistency of results, three quantification methods were used similar to Maeada et al. [41]. First, [^11^C]PiB uptake in the target region was divided by [^11^C]PiB uptake in the cerebellum as measured in a static 10 min-frame (20–30 min). Second, the tissue ratio methods as proposed by Ito et al. [71] were calculated. Third, the multilinear reference tissue model 2 (MRTM2, [45]) was fitted to cortex time-activity curves (TACs) (merged neocortex) after reduction of parameters by estimating individual efflux rate constants for [^11^C]PiB from the reference region (k2′) with the MRTM [45] and four regional cortical time-activity curves.

Parametric images of [^11^C]PiB retention (BP_ND_ maps (MRTM2)) were generated for four representative mice (Figure 2 and S1). For all analyses, the cerebellum was used as the reference region. To quantify the dynamic data, TACs with high initial time resolution (162 frames: 120×1 s, 24×10 s, 8×30 s, 10×300 s) were used (Figure 1).

### Biodistribution with [^11^C]PiB

#### Regional brain biodistribution

Identical conditions as used for in vivo PET imaging were implemented: Animals were kept under inhalation anesthesia (isoflurane) on a temperature-controlled heating pad (36°C) until death. All animals were killed by decapitation at 30 min p.i.. The entire brain was taken out and cut along the median sagittal line. One half was dissected into: 1.) olfactory bulb including ventral olfactory regions towards the olfactory tubercle, 2.) cerebellum, 3.) cortex and 4.) the remaining brain structures (diencephalon and midbrain). The other half of the brain was rapidly frozen for histology. Radioactivity in weighed tissues was determined using an automatic NaI(Tl) well-type γ-detector (Wallac 1480-011 Automatic Gamma Counter, PerkinElmer, Waltham,MA, USA), related to a standard and used for calculation of the injected dose per gram tissue (% ID/g).

A 30 min PET scan was acquired for all animals from the tgtg-old group immediately before biodistribution and ELISA assays of their brain tissues.

#### Cranial biodistribution of [^11^C]PiB

Additional to regional brain biodistribution, cranial organs were dissected as some of them are positioned very closely to frontal regions of the brain. Individual uptake behavior of the submandibular gland, sublingual gland, parotid gland, eyeballs (without muscles and optic nerve), internal lacrimal gland and harderian gland were assessed (Figure 3).

### Tissue processing

#### Genotyping

The tails of mice were preserved and deep-frozen at −75°C until processing. The genotype of all mice was re-evaluated and confirmed with quantitative PCR (qPCR) (Willuweit et al., personal communication).

#### Mouse brain for histology and autoradiography

Brain tissue was generally preserved, rapidly frozen in fine-crushed dry ice and stored air-tight at −75°C. For histological analyses, whole or half brains were cut on a Leica CM3050S cryostat (Leica Microsystems, Nussloch, Germany). Frozen sections were mounted on dilute poly-L-lysine hydrobromide coated (mol wt >300.000, (1∶50) 0.01% ^w^/_v_ in water) microscopy slides.

Based on the histopathological data of the transgenic animal model [31] we expected the highest plaque load in anterior cortex, medium plaque load in olfactory bulb and no plaque deposition in the cerebellum. To be able to correlate pathology and imaging findings in cortical and cerebellar regions within every single slice, we chose a cut level close to the horizontal sections in the Paxinos atlas [68].

About 120 sections with 10 µm thickness were mounted on about 40 slides. Slices were positioned to show three to four different cut levels on each slide, about 0.5 mm apart. About 5 of these slides were immediately stained with a thionin preparation for anatomical orientation within the slide sequence. After drying at ambient conditions the remaining slides were stored at −75°C until assayed.

#### Mouse brain tissue for quantitation of Aβ protein levels and PiB retention

The brains of mice from the major study groups (tg-old, tgtg-young, tgtg-old, ctl-old) were split into half and the cerebellum of each side was taken off as in Willuweit et al. [31] by cutting through along the coronal plane between the superior and inferior colliculus. All parts of the brain were stored at −70°C. All forebrains (right side without olfactory system) of the eight transgenic animals went through ELISA analysis. From each group, one representative sample was selected for subsequent radioligand binding assay with [^3^H]PiB.

#### Mouse cranium for [^3^H]PiB ex vivo autoradiography

To assess extracerebral uptake of [^3^H]PiB ex vivo the general protocol was slightly modified. Animals were killed at 30 min p.i.. The whole guillotined mouse heads were skinned such that external head glands were preserved at their natural positions. Upper and lower teeth were taken out. Air in nasal and oral cavities and nasal sinuses was displaced with wallpaper paste by intranasal lavage via a 26 Gauge IV cannula. The heads were deep-frozen at −75°C in a full wallpaper paste surrounding and cut on a Leica CM3500 cryostat for large tissue blocks (Leica Microsystems, Nussloch, Germany). Horizontal sections with 15 µm thickness were mounted on highly transparent cellulose-acetate tape. They were dried in the cryostat for 2 days and at ambient conditions for another 2 days. The sections on tape were mounted on microscopy slides and stored at −75°C until autoradiography.

#### Tissue homogenization of human and mouse brain

Tissue homogenization was performed to conform to both the protocol for ELISA analysis [31] and the radioligand binding assay [33].

The frozen mouse hemi-forebrains (50 mg/ml) and human brain samples (100 mg/ml) for the radioligand binding assay were first prepared in tissue homogenization buffer [33] (20 mM Tris base, 1 mM EDTA, 1 mM EGTA, cOmplete Protease Inhibitor Cocktail (Roche Applied Science, Mannheim, Germany)) using a 30 ml hand glass homogenizer (Dounce type, tight fit) (Sartorius Stedim Biotech, Göttingen, Germany) which was used for subsequent ELISA analysis. The stocks were then supplemented with 250 mM sucrose and stored at −70°C for later tracer binding analysis. All other tissue samples were processed for ELISA as reported by Willuweit et al.

### Autoradiography

The animals in the major subgroup of all study groups (Table 1) received [^3^H]PiB (specific activity: 2.78 TBq/mmol, radiochemical purity >97%) for ex vivo autoradiographical assessment of brain distribution of the tracer 9.3±1.7 hr after the [^11^C]PiB injection using the same injection protocol as in PET. After induction of isoflurane anesthesia, 6.95±0.81 MBq [^3^H]PiB was injected and flushed with isotonic saline through the catheter system such that the total applied volume was 0.21±0.02 ml. Once the [^3^H]PiB was applied intravenously, anesthesia was immediately switched to peritoneal antagonisable triple anesthesia (MMF) for MR scanning as explained above. Following the MR imaging procedure, the animals were guillotined at 62±2 min p.i., the full brain was removed within 8±2 min post-mortem, rapidly frozen in fine-crushed dry ice and stored at −75°C until autoradiographical data acquisition.

A total of 64 slides from 24 animals of the study collective were measured (Table 1). A minimum of 2 slides with at least three whole, or alternatively, at least four half horizontal sections were measured for each animal from the transgenic study groups and the old control group. 10 representative animals (6 transgenic and 4 controls) of the 24 were also measured in the digital autographical modality for validation purposes, providing 44 cortical and 43 cerebellar regional measurements. Sections from all animals were measured on tritium plates, providing 204 observations of the neocortical region and 195 observations of the cerebellar region.

#### Digital autoradiography

Digital autoradiographic images with a field of view of 24×32 mm were taken with the M40 series of μ-Imager™ (Biospace lab, Paris, France) using 10×10 cm scintillating foils with 13±1.5 µm thickness (Applied Scintillation Technologies, Harlow, England). The resolution with tritium is 20 µm, for carbon-11 it is about 40 µm, the detection threshold for tritium is 0.4 cpm/mm^2^, for carbon-11 it is 0.7 cpm/mm2 and the smallest pixel size is 1 µm. Instrument acquisition was controlled with μ-Acquisition software. Data was exported with β Vision+ software (both by Biospace lab). A coregistered optical image was taken with every scan.

Animals from the combined multi-modal subgroups were measured in dual-label mode after injection of a cocktail of [^11^C]PiB and [^3^H]PiB (Figure 6). Individual isotope signals were seperated with an automated algorithm.

#### Tritium plate autoradiography

A total of 50 slides with deep frozen CNS sections were dried in ambient air for 60 min and exposed under two halves of a large storage phosphor screen BAS-IP TR 2040 E (GE Healthcare Lifesciences, Freiburg, Germany). Then, the two plates were scanned with a CR35 Bio (Raytest, Straubenhardt, Germany) in sensitive 25 µm resolution mode. Scanning and data export was performed with AIDA (Raytest).

Validity and reliability of quantification results as measured by the tritium plate method was tested by measuring all samples from digital autoradiography on the tritium plates as well. At least two slides per transgenic animal containing three to four whole brain or four to five half brain sections were acquired (Table 1).

#### Quantification of [^3^H]PiB retention on autoradiographs

Lossless export of raw acquisition data to 16-bit grayscale TIFF images was executed with the software packages from the imaging device manufacturers (BetaVision (Biospace), AIDA Image Analyzer (Raytest)) for subsequent processing, analysis and finishing in Adobe Photoshop CS5 Extended (PS5) for Mac (Adobe Systems Inc., San Jose, USA).

Regions for analysis (forebrain, neocortex and cerebellum) were segmented in alpha channels of PS5 with neighboring Thioflavin S-stained sections and the Paxinos atlas as anatomical reference [68]. Integrated densities per region area were measured after background subtraction and used for ROI ratio analyses [72]. Equality and validity of results was confirmed by measuring samples of each group in NIH ImageJ, BetaVision and AIDA and by correlating the results from digital and tritium plate autoradiography.

Color tables for image presentation were imported from NIH ImageJ.

### Neurohistological staining

#### Thionin fast nuclear stain (Nissl)

Fresh tissue mouse brain sections were dried in ambient air for 15 min, immersed in 0.05% (^w^/_v_) thionin acetate in a buffer of 0.1 M acetic acid and 0.1 M potassium acetate for 4 min, mounted in Roti-Histokitt (Carl Roth, Karlsruhe, Germany) and protected with coverslips for rapid anatomical orientation within the set of histological material for each animal.

#### Thioflavin S staining

Fluorescent staining with Thioflavin S for frozen material was performed similar as reported by Willuweit et al. [31]. Deep frozen mouse brain sections were dried in ambient air for 15 min, immersion-fixed in ice-cold 4% (^w^/_v_) paraformaldehyde (Carl Roth, Karlsruhe, Germany) for 20 min. Sections were equilibrated in water twice for 2 min. Thioflavin S was dissolved at 1% (^w^/_v_) in water, and the solution was filtered. Sections were immersed in 1% Thioflavin S for 30 min at RT and kept dark, rinsed twice for 2 min in water, and differentiated in two changes of 80% ethanol (5 min and 1 min), washed in three changes of water (2 min each) and mounted in ProLong Gold antifade mounting medium (Invitrogen, Karlsruhe, Germany) with coverslips. Thioflavin S staining of all sections for Aβ plaque quantification was performed in a single staining procedure to ensure best possible comparability.

#### Aβ40 and Aβ42 Immunohistochemistry

Simultaneous double immunofluorescence for frozen material was performed similar to a staining protocol available in the Abcam (Abcam plc, Cambridge, UK) online protocol database [73] with slight modifications. Deep frozen mouse brain sections were dried in ambient air for 15 min, immersion-fixed in ice-cold 4% (^w^/_v_) paraformaldehyde (Carl Roth, Karlsruhe, Germany) for 10 min. Sections were equilibrated in three changes of 1% PBS (1 min each), permeabilized with 0.25% Triton X-100 in PBS for 10 min and washed in three changes of PBS (5 min each). They were then blocked with 10% normal donkey serum in PBS for 45 min, washed quickly in three changes of PBS-Tween20 (0.05%) and probed with the two primary antibodies (G2-10 (Merck Millipore, Schwalbach, Germany) and AB3 (Araclon Biotech, Zaragoza, Spain)) diluted in 1% BSA/PBS-Tween20 over night at 8°C. Afterwards, they were washed in three changes of PBS (5 min each) and incubated with two fluorophore-conjugated secondary antibodies (A488-D-Rb (Invitrogen, Karlsruhe, Germany) and Cy5-D-Ms (Jackson ImmunoResearch, Suffolk, UK) in 1% BSA/PBS for 2 hours. After three washes in PBS (5 min each), nuclei were stained by adding 0.5 µM DAPI (Sigma, Schnelldorf, Germany) for 1 min. After the final washing steps the tissue was coverslip-mounted with ProLong Gold Antifade mounting medium (Invitrogen/Molecular Probes, Darmstadt, Germany). Double anti-Aβ staining of all sections for Aβ plaque quantification was performed in a single staining procedure to ensure best possible comparability.

### Microscopy

Fluorescence microscopy for Acapella 2.0 analysis was performed as reported previously by Willuweit et al. [31]. Briefly, digital micrographs were acquired using a BX51 microscope (Olympus, Hamburg, Germany) with a ColorView II charge-coupled display (CCD) camera (Soft Imaging System, Olympus, Münster, Germany). The micrographs of horizontal mouse brain sections were recorded through a 2× objective followed by a 0.5× TV adaptor.

Entire-view high-resolution MosaiX pictures of horizontal mouse brain sections were created with an AxioImager Z.1 microscope (Carl Zeiss Microimaging, Munich, Germany) on a Zeiss CAN-Bus motor stage (Merzhäuser, Germany) using a 20×/0.5 M27 EC Plan-Neofluar Zeiss lens and Zeiss filter sets no. 49 (DAPI), no. 38 (HE Green Fluorescent Protein), no. 43 (HE DsRed), no. 47 (HE Cyan Fluorescent Protein) and no. 50 (Cy5). Micrographs were acquired with an AxioCam MRm Rev. 3.0 (Carl Zeiss Microimaging, Munich, Germany) camera. Data acquisition was controlled with AxioVision 4.8.1 and conversion of very large tiled MosaiX images to single 16-bit grayscale TIFF files per channel was performed with AxioVision 4.8.2 SE64.

### Histological quantification of relative Aβ plaque burden and plaque size distribution

Image analysis was performed basically as described in our recent study [31] with a few modifications. Digital images were evaluated with Acapella™ 2.0 data analysis software (PerkinElmer, Hamburg, Germany) using an updated plaque quantification script for specific and sensitive recognition of individual plaques and for quantitative assessment of relative plaque load.

Aβ plaque burden was quantified using two different fluorescent modalities: Thioflavin S-stained sections were analyzed using the single channel method reported previously [31]. Double anti-Aβ40 and anti-Aβ42 immunostained sections were analyzed using two channels simultaneously after skew correction.

Regions of interest (neocortex, thalamus and cerebellum) were defined by manual segmentation in accordance with the anatomical delineations given by Paxinos and Franklin [68] using Adobe Photoshop CS5 Extended for Mac (Adobe Systems Inc., San Jose, CA, USA).

For the Aβ40 channel, observations of smallest plaques were excluded in order to control for mouse-on-mouse non-specificity of the primary antibody. The validity and reliability of this approach was successfully tested against the anti-Aβ42 and Thioflavin S channels.

All sections contained major portions of the neocortex and cerebellum. The thalamus could be delineated in the majority of these sections. Measurements provided total plaque area per defined regional area (relative total plaque burden) and counts for individual plaque sizes (plaque size distribution).

Right-left comparisons were performed with thirteen animals of the study collective (tg-old (3), tgtg-young (3), ctl-young (3) and ctl-old (4)) and the data showed no differences.

Hence, 643 cortical and 411 thalamical regions were measured on horizontal Thioflavin S stained sections (Table 1). Analogously, 158 cortical and 106 thalamical regions were measured on nearby or neighboring horizontal anti-Aβ40/42 stained sections.

To visualize plaque size distribution in neocortex and thalamus among the study groups with Aβ deposits, measured plaque areas were considered as circles and categorized according to their radii. To analyze the differences between groups and regions, we histogrammed the individual plaque sizes for each transgenic group and region, estimated Epanechnikov kernel density functions and performed two-sample Kolmogorov-Smirnov tests to test whether the two underlying one-dimensional probability distributions differ.

### Aβ protein quantification with ELISA

Differential extraction of soluble and insoluble Aβ_x–40_ and Aβ_x–42_ in human and mouse brain (Table 1) was performed as described in detail previously [68] with slight modifications. Briefly, brain homogenates were centrifuged at 53000 rpm for 30 min at 4°C. Supernatant and pellets were stored at −80°C. Pellets were resuspended in the same volume of 70% formic acid, kept on ice for 30 min and centrifuged likewise. Resulting supernatants were neutralized with 19 volumes of 1 M Tris pH = 11.3. Aβ peptides were quantified in supernatants of both extractions using commercially available ELISA kits (EZBRAIN40 (G2-10 clone) and EZBRAIN42 (G2-13 clone), Merck Millipore, Schwalbach, Germany). Results are expressed as picogram Aβ per microgram tissue wet weight.

### Combined multi-modal experiment

To gain a large spectrum of information across the various modalities from a single animal, a combined multi-modal in vivo, ex vivo and in vitro experiment with co-administration of [^11^C]PiB and [^3^H]PiB was performed for four animals of the young study group (2 tgtg-young and 2 ctl-young) (Figure 6, Table 4)).

After each animal had gone through a CT scan in the docked PET/CT system, a cocktail of [^11^C]PiB and [^3^H]PiB was injected and a PET image taken over 30 min. Then, the radioactivity of the whole animal was measured in a Capintec dose calibrator, the animal killed by decapitation, the whole brain taken out and blood drawn for biodistribution and radioactivity measurement. One half of the brain was dissected for regional brain biodistribution (olfactory system, telencephalon, cerebellum and remaining brain structures (diencephalon, midbrain)) and deep-frozen immediately after gamma counting for later ELISA analysis of soluble and insoluble Aβ_x–40_ and Aβ_x–42_. The other half was frozen on dry ice and rapidly cut on a cryostat for dual-isotope digital autoradiography (started around 1 hour p.i.) with subsequent automated separation of isotope signals, and for later histological processing for microscopy and histological Aβ plaque quantification with Thiofavin S, anti-Aβ40 and anti-Aβ42. Injected doses of [^11^C]PiB in these two subgroups was higher than in the rest of the study collective (see Table 1) to retain a sufficient signal for [^11^C]PiB autoradiography while specific activities were remaining on the common clinical routine level.

### Radioligand saturation binding assay with [^3^H]PiB

Linearity of [^3^H]PiB binding was confirmed using the huAD-C sample for a range of tracer (0.2 µM to 80 µM) and target (20 to 2000 µg/ml) concentrations and tracer incubation times (1 h to 9 h)). Test-retest studies with the biological tissue (huAD-C) revealed robustness of the method with high reliability (Figure S9).

All brain homogenates were diluted in PBE buffer (10 mM dibasic sodium phosphate, 1 mM EDTA, 10% EtOH) [74] to a final concentration of 1000 µg/ml. Synthetic human Aβ_1–40_ fibrils (EZBioLab, Carmel, IN, USA) were prepared as described by Lockhart et al. (0.5 mg/ml, pH 7.4, 200 rpm at 37°C for 48 h) and were used as positive control at a final concentration of 10 µg/ml (Figure S8).

The fixed concentration of brain homogenate was titrated against twelve concentrations of [^3^H]PiB (0.2 nM to 48 nM, specific activity 3.15 GBq/µmol, Quotient Bioresearch, Fordham, UK) for 3 h at 21°C on a flat shaker at 240 rpm (IKA-Werke, Staufen, Germany). Nonspecific binding was determined in the presence of 3 µM unlabeled PiB (ABX, Radeberg, Germany) including 1 h preincubation. Each tissue sample was deployed on two 96-well cell culture plates (Greiner Bio-One, Frickenhausen, Germany) to a final reaction volume of 280 µl per well using 8-channel electronic pipettes (Mettler Toledo, Giessen, Germany) giving 24 octuples of data points per sample.

The bound and free fractions were seperated by vacuum filtration through 0.3% polyethyleneimine-pretreated [75] GF/B glass filtermats (GE Healthcare/Whatman, Dassel, Germany) using a semi-automated Harvester 96 Mach II M (Tomtec, Hamden, CT, USA). A constant automated 5-cycle pulse-wash program (optimized for flow rate and volume) guaranteed stable harvesting with 1.2 ml PBE washing per well at 2 psi.

Filters were cut and incubated in Aquasafe300plus scintillator (Zinsser Analytic, Frankfurt, Germany) for 36 h before counting in a Wallac WinSpectral 1414 liquid scintillation counter (PerkinElmer, Rodgau, Germany). Free tracer (octuples of all twelve [^3^H]PiB concentrations) and background were measured in every experiment.

The specific binding signal under these assay conditions was between 56% (human) and 91% (synthetic protein). Data were analyzed using GraphPad Prism version 5.0 d for Mac (GraphPad Software, La Jolla, CA, USA) to estimate the apparent dissociation constant (K_d_), the maximal number of binding sites (B_max_) and the in vitro binding potential (BP) using the one-site and two-site global analysis models.

To facilitate comparison among the in vitro [^3^H]PiB binding data and to provide a measure for comparison with in vivo PET data, we converted our results to in vitro binding potential (BP) [76], [77] similar to Klunk et al [33]. B_max_ was converted from femtomoles of [^3^H]PiB per milligram of brain to nanomolar units assuming that one gram of brain equals one milliliter of brain volume (i.e. 1 fmol/mg = 1 pmol/g≡1 pmol/ml (of brain) = 1 nmol/l = 1 nM). Division by K_d_ in nanomolar units directly results in BP.

### Statistical analysis

For statistical analysis and graphical output, all data tables were transformed from Microsoft Excel for Mac 2011 to Stata format with Stat/Transfer 10 (Circle Systems, Seattle, WA, USA) for analysis in Stata/IC 11.2 for Mac (Stata Corp., College Station, TX, USA) if not noted otherwise.

Two-sided t-tests with unequal variances were used to test for differences between groups. Significance level was set to 5% if not specified otherwise.

All reported correlations are pair-wise Pearson correlation coefficients (*r*).

## Supporting Information

Figure S1 **[^11^C]PiB PET binding potential maps for mouse brain.** PET binding potential maps for [^11^C]PiB in Alzheimer mouse brains and healthy control brain showing individual data from the complete orthogonal PET/MR image data (BP_ND_, MRTM2) corresponding to Figure 2. (**A**) 23 month old female hemizygous APP/PS1 mouse, (**B**) 9 month old female homozygous APP/PS1 mouse, (**C**) 21 month old female homozygous APP/PS1 mouse, (**D**) 23 month old female C57BL/6J control mouse. PET color look-up-table is *UCLA* (Pmod). Arrowheads (*gray*) indicate slice positions. The shown coordinates are identical to those shown in Figure 1. For horizontal slices (corresponding to Paxinos mouse brain atlas) they are Bregma −1.90 mm, for coronal Bregma −0.10 mm and for sagittal 0.65 mm lateral (right side).
(TIF)

Figure S2 **[^11^C]PiB/[^18^F]FDG sequential PET in healthy control.** For reliable image co-registration (Figure S3) and evaluation of extracerebral tracer uptake (Figure 3), several transgenic and control animals were additionally injected with [^18^F]FDG immediately after their [^11^C]PiB scan via the other lateral tail vein and without moving the animals. Shown, here, are the orthogonal views (**A**) at the same locations as in the other figures and the horizontal views (**B**) from top to bottom (1 mm apart) of an animal from the ctl-old study group which was scanned for 120 min with [^11^C]PiB (60 min) (*red*) and [^18^F]FDG (60 min) (*green*) without being moved in the scanner and which received an MR scan, the same day. The [^18^F]FDG image was co-registered to the MR scan and the resulting transformation matrix applied to the [^11^C]PiB image. The static 30 min frames of the last halves of each scan are shown in combination without any manual co-registration among these datasets. Co-localization (*yellow*) shows that, in this animal, the harderian glands have the largest contribution to unspecific [^11^C]PiB uptake in the eye cavities. Arrowheads (*gray*) in (A) indicate slice positions. The coordinates for horizontal slices (corresponding to Paxinos mouse brain atlas) are Bregma −1.90 mm, for coronal Bregma −0.10 mm and for sagittal 0.65 mm lateral (right side).
(TIF)

Figure S3 **Mouse brain PET/MRI image co-registration.** Principle of manual co-registration process as applied for all PET data of this study. Proximity of frontal cortex to extracerebral regions with high unspecific [^11^C]PiB retention as shown in Figure 3 requires precise co-registration for reliable PET analyses. Three co-registered image modalities are shown in each panel: PET template of early (1–4 min) radiotracer entrance (*red*), MRI template (*gray*) and cranial CT (*green*). CTs and MRIs are co-registered to Paxinos space along all axes. *Top row* (**A**): horizontal views from top to bottom (1.0 mm apart). *Middle row* (**B**): coronal views from nose to back of head (2.1 mm apart). *Bottom row* (**C**): sagittal views from median to left (1.4 mm apart).
(TIF)

Figure S4 **Volume-of-interest definition.** Volumes-of-interest (VOI) were defined on horizontal sections of mouse brain MRI template in Paxinos atlas space. Defined paired and non-paired neuroanatomical and cranial structures are cortex (neocortex (*magenta*) and hippocampus (*red*)), thalamus (*orange*), olfactory bulb (*lavender*), cerebellum (*yellow*), eyebulbs (*bright green*), harderian glands (*dark green*), nasal sinuses (*light blue*). The same region definition was used for autoradiographs and microscopic sections.
(TIF)

Figure S5 **PET test-retest results.** Three mice from the tgtg-old study group were scanned twice with the same PET imaging protocol. Scans were about one week apart. Time activity curves show dynamic neocortical (*red*) and cerebellar (*blue*) uptake during first (*dark*) and second (*light*) scan. Peak of cerebellar [^11^C]PiB uptake was taken as maximum for each scan. Middle panel shows how the injection during the retest scan was slower than during the test scan while the specific uptake tail of the curves approach each other. One retest scan (**C**) was for 30 min while all other scans were of 60 min duration. Binding potential values (BP_ND_) as estimated with MRTM2 are noted in each panel for test/retest scans.
(TIF)

Figure S6 **Time-activity curve averages per group.** Averaged neocortical (*red*) and cerebellar (*blue*) time-activity curves (TACs) for the major study groups. The behavior of individual TACs as shown in Figures 1 is also reflected in the groupwise behavior. Peak of cerebellar [^11^C]PiB uptake was taken as maximum for each animal. Vertical bars depict one standard deviation from group average. (**A**) tg-old, (**B**) tgtg-young, (**C**) tgtg-old and (**D**) ctl-old.
(TIF)

Figure S7 **Thioflavin S and Aβ40/42 antibodies for histological quantification of Aβ plaques.** Aβ plaque burden was analyzed on histological sections stained with Thioflavin S and double immunofluorescence against Aβ40 and Aβ42 by applying a semi-automatic imaging algorithm. All animals were analyzed in PET, before. Scatter plot matrix showing individual relative plaque areas (%) for neocortex of the transgenic study groups in all three staining modalities including the compound signal of the Aβ40/42 antibodies. The results show how plaque area quantification with Thioflavin S tightly correlates to the specific antibodies. Hence, it was used representatively in Figure 7. Pairwise correlation coefficients (*r*) for each pair of modalities are noted in each panel. The study groups in the scatter matrix are identified by color: tg-old (*orange*), tgtg-young (*yellow*) and tgtg-old (*red*).
(TIF)

Figure S8 **Radioligand binding assay for synthetic Aβ40 fibrils.** Positive synthetic protein control for radioligand saturation binding assay with [^3^H]PiB to human and mouse brain tissue. (**A**) Total and nonspecific binding data octuples for synthetic Aβ_1–40_ fibrils. Solid curves show nonlinear fits with two-site model. Dashed lines describe 95% confidence bands around the fit. (**B**) Specific binding curve for [^3^H]PiB to Aβ_1–40_ fibrils as a result from total and nonspecific as shown in (A). (**C**) Scatchard graph created with data shown in (B). Each data point is derived from the mean value of the original data octuples. (**D**) Semilogarithmic representation of the specific binding data as seen in (B) to show infliction points. Therefore, more appropriate estimates are yielded by fitting the global two binding sites model. B_max_, K_d_ and BP values for the low- and high-affinity binding sites of this dataset are noted at the bottom.
(TIF)

Figure S9 **Binding assay test-retest results.** [^3^H]PiB binding to the severely affected human AD tissue sample (huAD-C) was repeated twice to test robustness of the method for biological material. Solid curves show nonlinear regression fits with single binding site model. Dashed lines describe 95% confidence bands around the fit. Binding data yielded from the single site model are noted in the panel for each experimental run.
(TIF)

Figure S10 **Cross-validation of PET with other modalities.** Summary of the major experimental results for the study collective as a scatter matrix. Correlation of in vivo [^11^C]PiB PET binding potential for mouse neocortex with relative neocortical [^3^H]PiB uptake in autoradiography, with relative neocortical Aβ plaque burden as stained by Thioflavin S and with insoluble Aβ_x–40_ and Aβ_x–42_ protein levels in forebrain (*left column*) as presented in Figure 7. Remaining scatter plots show robustness, consistency and validity of the cross-validation. Neocortex was used as the primary target region except for ELISA (Aβ_x–40_ and Aβ_x–42_ protein levels) where the whole forebrain was used according to previous protocols. Data across the modalities was acquired from tissue of the same animals (as shown in Table 1). Individual animals are identified by their unique number code within their study group. The coloring of study groups in the scatter plots shows how each group is fully separated from each other. Color code: tg-old (*orange*), tgtg-young (*yellow*), tgtg-old (*red*) and ctl-old (*blue*). Pairwise correlation coefficients (*r*) for each pair of modalities are noted in each scatter plot. Histological quantification with Thioflavin S is used representatively for all histological quantification results because of its tight correlation with anti-Aβ40/42 as described in Figure S7. Here, the animals presented in Figures 1 and 2 are coded with #5 (tg-old), #5 (tgtg-young), #1 (tg-old) and #1 (ctl-old).
(TIF)

Table S1 **Robustness of PET results.** PET results shown as averages for the major study groups with neocortex as target region and cerebellum as reference region. Results were tested for differences between groups corresponding to the staging of Aβ load in these groups (i.e. p-value for difference to group below). Here, significance level is set to 1%. Names of study groups correspond to Table 1.
(PDF)
