# Supplementary figures and images for: Small-Animal PET Imaging of Amyloid-Beta Plaques with [11C]PiB and Its Multi-Modal Validation in an APP/PS1 Mouse Model of Alzheimer's Disease
Source: PLoS One. 2012 Mar 9;7(3):e31310. doi: 10.1371/journal.pone.0031310 (PMC3302888; doi:10.1371/journal.pone.0031310)

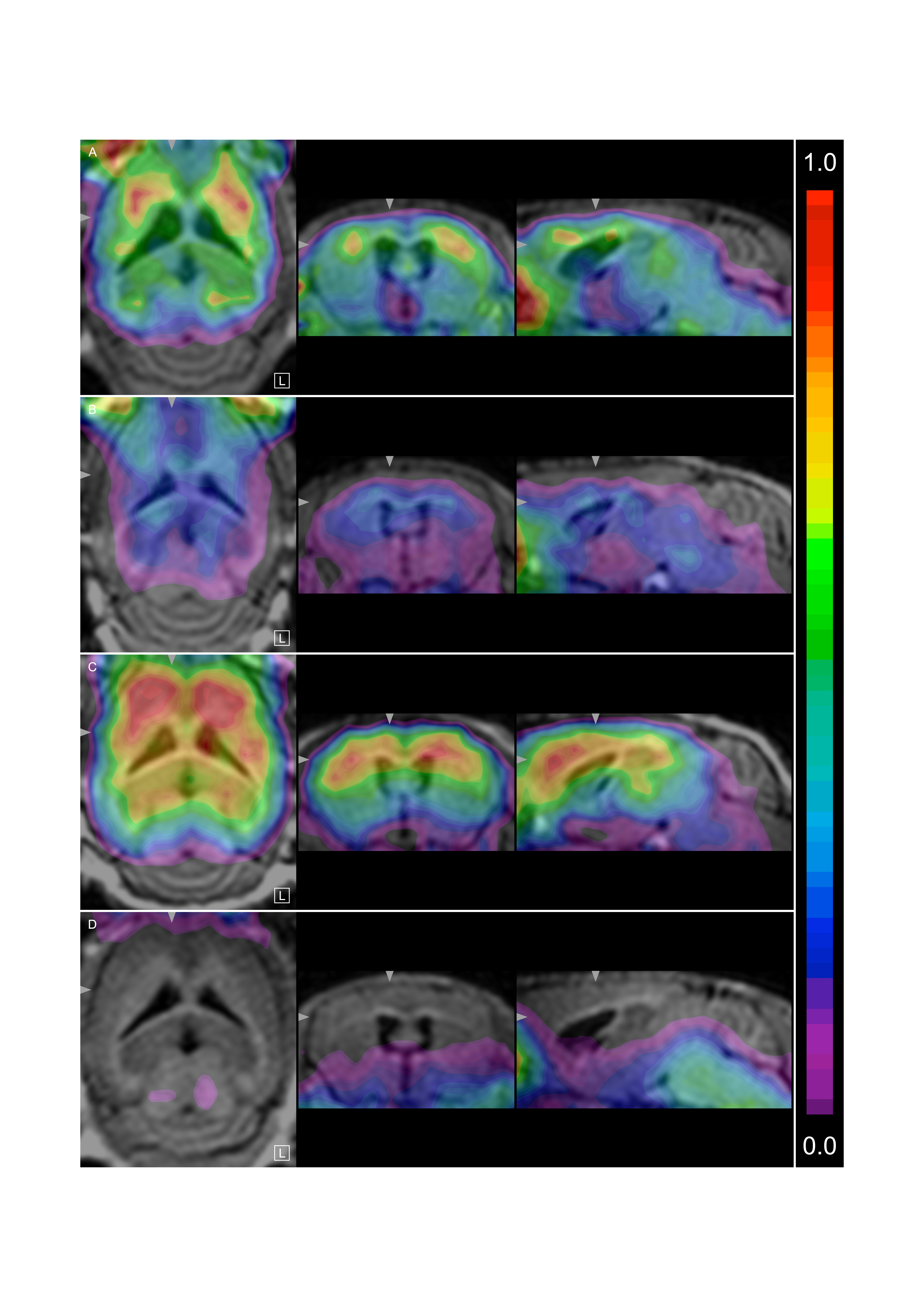

Supplement: Figure S1 — [11C]PiB PET binding potential maps for mouse brain. PET binding potential maps for [11C]PiB in Alzheimer mouse brains and healthy control brain showing individual data from the complete orthogonal PET/MR image data (BPND, MRTM2) corresponding to Figure 2. (A) 23 month old female hemizygous APP/PS1 mouse, (B) 9 month old female homozygous APP/PS1 mouse, (C) 21 month old female homozygous APP/PS1 mouse, (D) 23 month old female C57BL/6J control mouse. PET color look-up-table is UCLA (Pmod). Arrowheads (gray) indicate slice positions. The shown coordinates are identical to those shown in Figure 1. For horizontal slices (corresponding to Paxinos mouse brain atlas) they are Bregma −1.90 mm, for coronal Bregma −0.10 mm and for sagittal 0.65 mm lateral (right side). (TIF) [file pone.0031310.s001.tif]

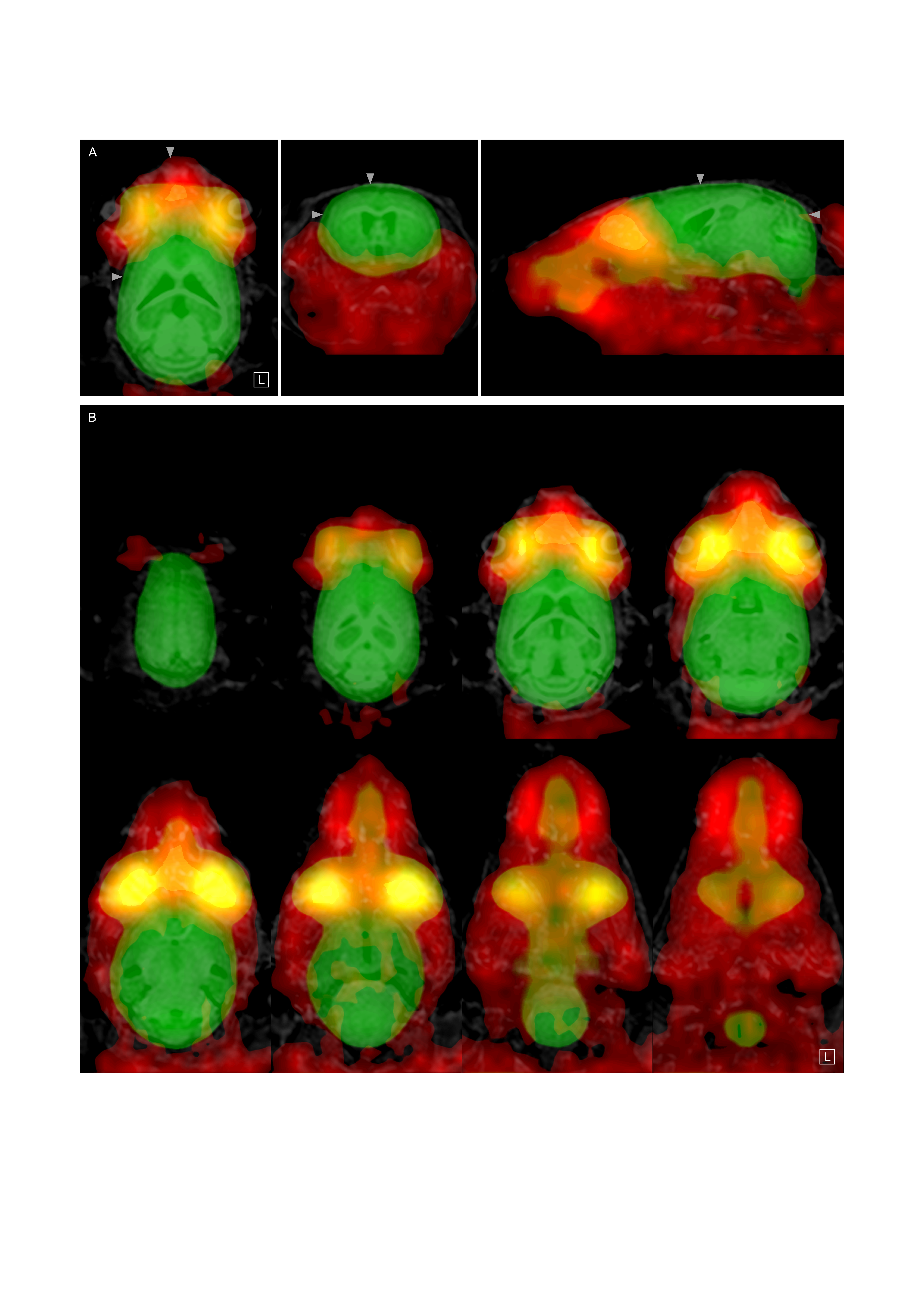

Supplement: Figure S2 — [11C]PiB/[18F]FDG sequential PET in healthy control. For reliable image co-registration (Figure S3) and evaluation of extracerebral tracer uptake (Figure 3), several transgenic and control animals were additionally injected with [18F]FDG immediately after their [11C]PiB scan via the other lateral tail vein and without moving the animals. Shown, here, are the orthogonal views (A) at the same locations as in the other figures and the horizontal views (B) from top to bottom (1 mm apart) of an animal from the ctl-old study group which was scanned for 120 min with [11C]PiB (60 min) (red) and [18F]FDG (60 min) (green) without being moved in the scanner and which received an MR scan, the same day. The [18F]FDG image was co-registered to the MR scan and the resulting transformation matrix applied to the [11C]PiB image. The static 30 min frames of the last halves of each scan are shown in combination without any manual co-registration among these datasets. Co-localization (yellow) shows that, in this animal, the harderian glands have the largest contribution to unspecific [11C]PiB uptake in the eye cavities. Arrowheads (gray) in (A) indicate slice positions. The coordinates for horizontal slices (corresponding to Paxinos mouse brain atlas) are Bregma −1.90 mm, for coronal Bregma −0.10 mm and for sagittal 0.65 mm lateral (right side). (TIF) [file pone.0031310.s002.tif]

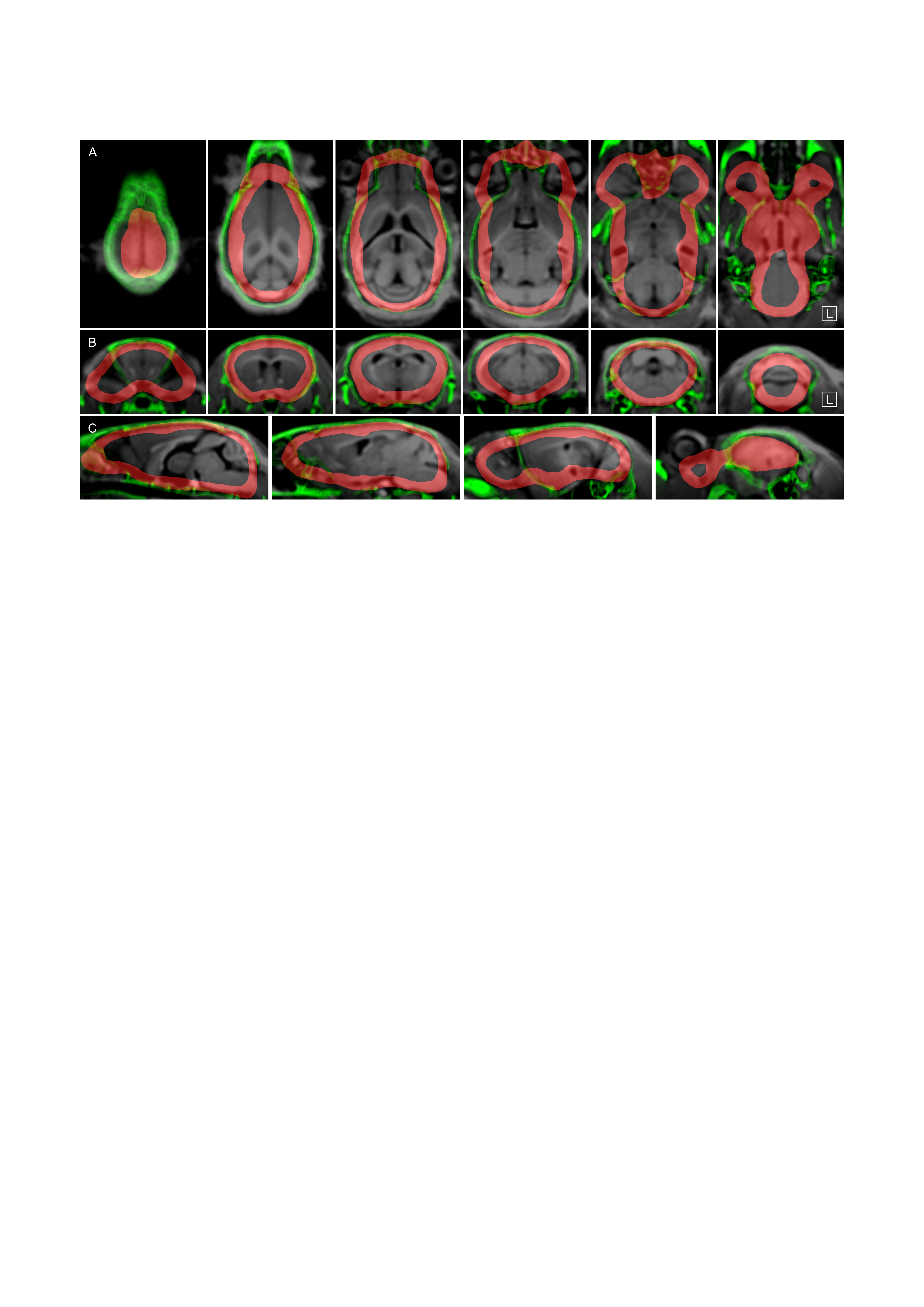

Supplement: Figure S3 — Mouse brain PET/MRI image co-registration. Principle of manual co-registration process as applied for all PET data of this study. Proximity of frontal cortex to extracerebral regions with high unspecific [11C]PiB retention as shown in Figure 3 requires precise co-registration for reliable PET analyses. Three co-registered image modalities are shown in each panel: PET template of early (1–4 min) radiotracer entrance (red), MRI template (gray) and cranial CT (green). CTs and MRIs are co-registered to Paxinos space along all axes. Top row (A): horizontal views from top to bottom (1.0 mm apart). Middle row (B): coronal views from nose to back of head (2.1 mm apart). Bottom row (C): sagittal views from median to left (1.4 mm apart). (TIF) [file pone.0031310.s003.tif]

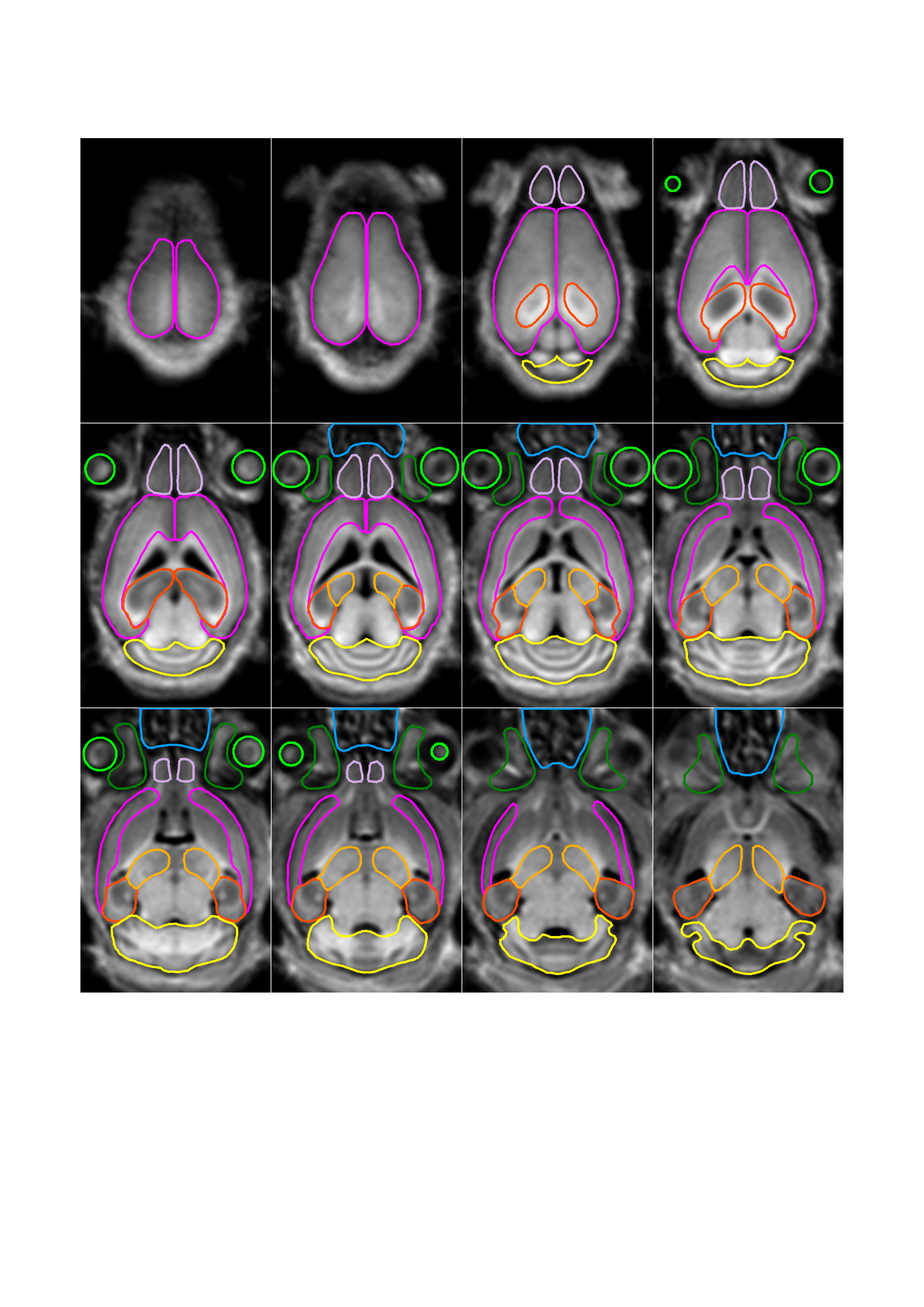

Supplement: Figure S4 — Volume-of-interest definition. Volumes-of-interest (VOI) were defined on horizontal sections of mouse brain MRI template in Paxinos atlas space. Defined paired and non-paired neuroanatomical and cranial structures are cortex (neocortex (magenta) and hippocampus (red)), thalamus (orange), olfactory bulb (lavender), cerebellum (yellow), eyebulbs (bright green), harderian glands (dark green), nasal sinuses (light blue). The same region definition was used for autoradiographs and microscopic sections. (TIF) [file pone.0031310.s004.tif]
