# Supplementary material for: Small-Animal PET Imaging of Amyloid-Beta Plaques with [11C]PiB and Its Multi-Modal Validation in an APP/PS1 Mouse Model of Alzheimer's Disease
Source: PLoS One. 2012 Mar 9;7(3):e31310. doi: 10.1371/journal.pone.0031310 (PMC3302888; doi:10.1371/journal.pone.0031310)
Supplement: Table S1 — Robustness of PET results. PET results shown as averages for the major study groups with neocortex as target region and cerebellum as reference region. Results were tested for differences between groups corresponding to the staging of Aβ load in these groups (i.e. p-value for difference to group below). Here, significance level is set to 1%. Names of study groups correspond to Table 1. (PDF) [file pone.0031310.s011.pdf]

**study group**	**BP_{ND}** **(MRTM2, Ichise et al., 2003)**		**tissue integral ratio** **(Ito et al., 1998)**		**target-to-reference ratio** **(20-30 min static)**	
	mean ± SD	p-value	mean ± SD	p-value	mean ± SD	p-value
**ctl-old** **(N = 5)**	-0.08 ± 0.01	< 0.001	0.0007 ± 0.0005	< 0.001	0.90 ± 0.02	< 0.001
**tgtg-young** **(N = 5)**	0.12 ± 0.04	0.001	0.0614 ± 0.0034	0.074	1.20 ± 0.06	0.002
**tg-old** **(N = 5)**	0.28 ± 0.06	< 0.001	0.0948 ± 0.0310	< 0.001	1.38 ± 0.06	< 0.001
**tgtg-old** **(N = 7)**	0.51 ± 0.13		0.1747 ± 0.0240		1.68 ± 0.12	
